# Organizational Factors Determining Access to Reperfusion Therapies in Ischemic Stroke-Systematic Literature Review

**DOI:** 10.3390/ijerph192316357

**Published:** 2022-12-06

**Authors:** Ana Botelho, Jonathan Rios, Ana Paula Fidalgo, Eugénia Ferreira, Hipólito Nzwalo

**Affiliations:** 1 Faculty of Economy, University of Algarve, 8005-139 Faro, Portugal; 2 Department of Physical Medicine and Rehabilitation, Algarve Hospital University Center-Faro, 8000-386 Faro, Portugal; 3 Stroke Unit, Algarve Hospital University Center-Faro, 8000-386 Faro, Portugal; 4 Faculty of Medicine and Biomedical Sciences, University of Algarve, 8005-139 Faro, Portugal; 5 Algarve Biomedical Research Institute, 8005-139 Faro, Portugal

**Keywords:** ischemic stroke, thrombolysis, thrombectomy, endovascular treatment with access and delay

## Abstract

Background: After onset of acute ischemic stroke (AIS), there is a limited time window for delivering acute reperfusion therapies (ART) aiming to restore normal brain circulation. Despite its unequivocal benefits, the proportion of AIS patients receiving both types of ART, thrombolysis and thrombectomy, remains very low. The organization of a stroke care pathway is one of the main factors that determine timely access to ART. The knowledge on organizational factors influencing access to ART is sparce. Hence, we sought to systematize the existing data on the type and frequency of pre-hospital and in-hospital organizational factors that determine timely access to ART in patients with AIS. Methodology: Literature review on the frequency and type of organizational factors that determine access to ART after AIS. Pubmed and Scopus databases were the primary source of data. OpenGrey and Google Scholar were used for searching grey literature. Study quality analysis was based on the Newcastle-Ottawa Scale. Results: A total of 128 studies were included. The main pre-hospital factors associated with delay or access to ART were medical emergency activation practices, pre-notification routines, ambulance use and existence of local/regional-specific strategies to mitigate the impact of geographic distance between patient locations and Stroke Unit (SU). The most common intra-hospital factors studied were specific location of SU and brain imaging room within the hospital, and the existence and promotion of specific stroke treatment protocols. Most frequent factors associated with increased access ART were periodic public education, promotion of hospital pre-notification and specific pre- and intra-hospital stroke pathways. In specific urban areas, mobile stroke units were found to be valid options to increase timely access to ART. Conclusions: Implementation of different organizational factors and strategies can reduce time delays and increase the number of AIS patients receiving ART, with most of them being replicable in any context, and some in only very specific contexts.

## 1. Introduction

**Figure 1:**
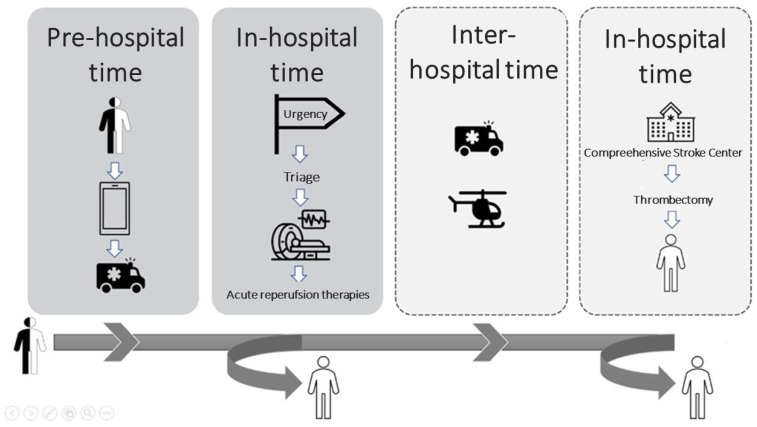
Possible trajectory of an ischemic stroke patient from initiation of treatment to reperfusion therapy.

Stroke is a major public health problem worldwide causing approximately 6.2 million deaths per year [1]. Of all acute ischemic stroke (AIS) types, those resulting from proximal occlusion of cervical or intracranial large vessels have the worst prognosis. Timely delivery of acute reperfusion therapies (ART) with thrombolysis in the first 4.5 h and/or thrombectomy up to 24 h is essential to restore normal brain circulation and prevent death and disability [2]. The odds ratio (OR) of an excellent outcome (free of disability) with thrombolysis compared with placebo decreases with treatment delays, from 2.8 when patients are treated between 0 and 90 min, to 1.6 for 91 to 180 min, 1.4 for 181 to 270 min, and 1.2 for 271 to 360 min [3]. The OR of better functional outcomes with thrombectomy also decreases with time, from 2.79 (95% CI, 1.96 to 3.98) in the first three hours to 1.98 (95% CI, 1.30 to 3.00) at 6 h [4]. Unfortunately, only 7.3% of all patients with acute ischemic stroke receive thrombolysis and only 1.9% undergo thrombectomy [5]. Several cognitive, clinical and socio-demographic factors contribute to delays or even access to the ART. From the onset of symptoms to hospital patient arrival (pre-hospital delay) as well from the moment of admission, usually in the emergency room (ED) to the final treatment decision (intrahospital delay) (Figure 1), organizational factors may determine timely access to ART [6].

Despite its critical importance to public health planification, systematic analysis of these factors has never been performed. Therefore, we decided to review the literature to gather information on organizational factors that determine timely access to ART.

## 2. Materials and Methods

Search Strategy: Pubmed and Scopus databases were used to search for relevant publications addressing organizational factors and strategies associated with timely access to ART using the following term associations: “ischemic stroke”, thrombolysis, thrombectomy, “endovascular treatment” with “access” and “delay”. We complemented this search by examining reference lists of the most relevant studies and the Open grey database (http://www.opengrey.eu/, accessed on 1 January 2021).

Criteria for Inclusion and Exclusion of Studies: Prospective and retrospective studies published after endovascular treatment approval for AIS (2015), excluding studies after 31 December 2020 to minimize the contribution of the COVID-19 pandemic containing information on one of the following domains under analysis: pre-hospital organizational (population education, emergency stuff training, emergency activation, ambulance use, existence of specific stroke code protocols, mobile stroke unit, and telemedicine/telestroke), intra-hospital (hospital pre-notification, stroke unit, protocols, specific imaging protocols, pre-notification of the neuroradiology team, telemedicine/telestroke), and inter-hospital (transport). Conference or seminar’s abstracts and/or studies with unclear inclusion criteria, studies based on selective or convenience sampling and non-English publications were excluded.

Data Extraction: Titles and abstracts were independently verified by 2 investigators (AB, JS). Disagreements regarding the inclusion of specific studies were resolved by a third investigator. Strengthening the Reporting of Observational studies in Epidemiology (STROBE) checklist for systematic reviews was used to guide data extraction. The verification of the duplication of studies was performed automatically using the Mendeley bibliographic reference management system.

The identified and rejected studies were recorded in a separate database, documenting the main reason for their exclusion (Appendix A-Table A1).

Data Synthesis: The data were analyzed descriptively. No meta-analysis was anticipated or performed due to the expected and marked heterogeneity and methodological variability of the studies. Study quality was assessed using “The Newcastle-Ottawa Scale (NOS)” instrument [7] (Appendix A-Table A2).

## 3. Results

**Figure 2:**
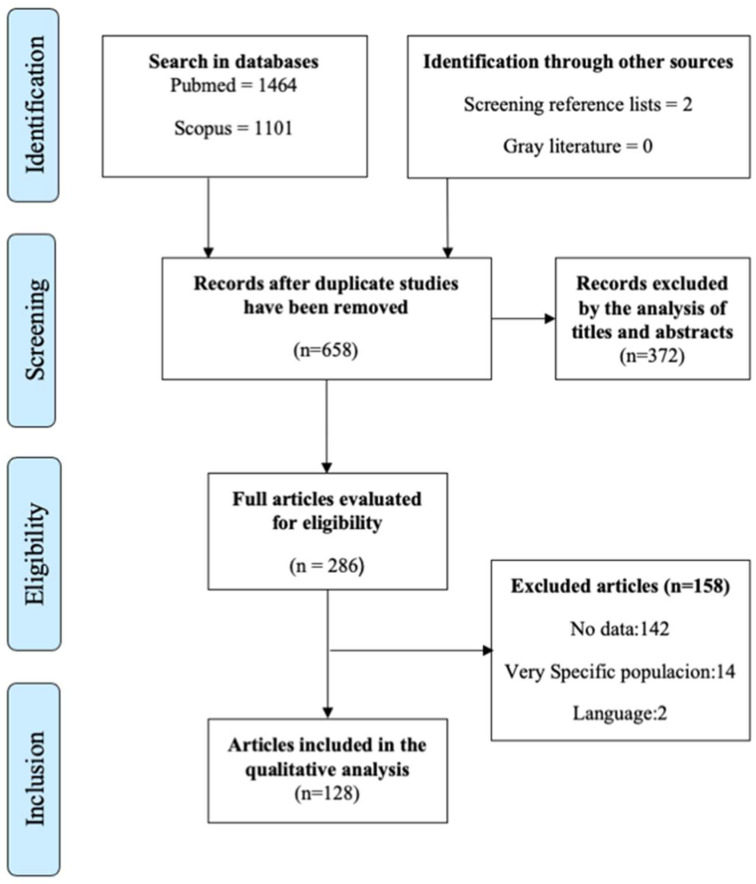
PRISMA-P flowchart of the process of inclusion of studies in the systematic review.

We identified a total of 1464 (Pubmed) and 1101 (Scopus) publications using the predefined searching criteria. Preferred Reporting Items for Systematic Reviews and Meta-Analyses (PRISMA) flowchart diagram (Figure 2) resumes the selection and inclusion process.

**Figure 3:**
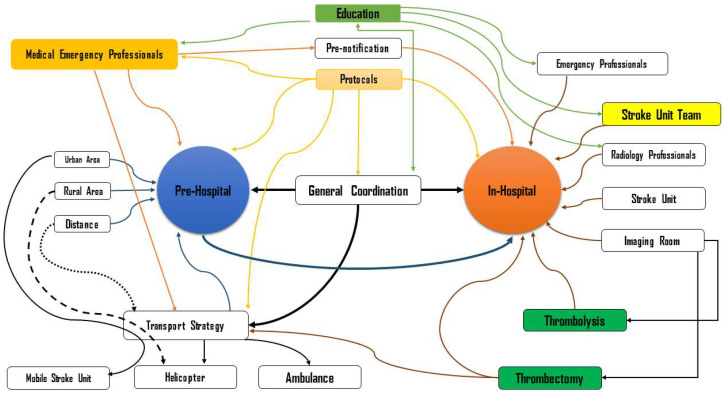
Pre-hospital and in-hospital factors.

The number of studies after deduplication was 658. A total of 286 papers were selected as relevant for complete text evaluation, after which, a further 158 studies were excluded (Appendix A-Table A3). The three main reasons for exclusion were no data (*n* = 142/89.8%), very specific population (*n* = 14/8.86%) and language (*n* = 2/1.26%). Figure 3 summarizes the organizational factors identified in the systematic review.

### 3.1. Pre-Hospital Organizational Factors

We identified 67 studies addressing pre-hospital organizational factors associated with delay or access to ART (Appendix A-Table A3). Activation of stroke code, pre-hospital notification and use of pre-hospital emergency services [8,9,10,11,12,13,14,15,16,17,18,19,20,21,22,23,24,25,26,27,28], mobile stroke units [24,25,26,27,28,29,30,31,32], implementation of specific electronic apps to insert and share clinical data with the hospital stuff [33], use of ambulance [34,35,36,37,38,39,40,41,42,43,44,45,46], telestroke [47,48], implementation of specific pre-hospital protocols [49,50], education campaigns [48,49,50,51,52,53], geographical division of areas based on population agglomerates and distance from the SU to stratify resources allocation, including the pre-hospital emergency care [50,54,55]. Mobile SUs with ambulances equipped with brain computed axial tomography and thrombolysis capability reduced treatment delays and increased the number of patients receiving alteplase [24,25,27,28,29,30,31,32]. Mobile SUs were shown to be cost-effective in areas with high population density [25,28]. Pre-hospital screening scales to select patients at high risk of large vessel occlusion and direct transfer to large centers with endovascular or thrombectomy treatment [55,56,57,58,59,60] also increased the number of patients with AIS receiving thrombectomy. This approach dictates modification of the current stroke patients transport paradigm from the drip and ship model (transport to the closest thrombolysis center) to the motherboard model (direct transport to a thrombectomy center) [55,56,57,58,59,60].

### 3.2. Intra-Hospital Organizational Factors

We found 49 studies containing information on intra-hospitalar organizational factors associated with delays or access to ART (Appendix A-Table A3). Training emergency room professionals [61,62,63,64,65,66], promotion of specific stroke code protocols [30,67,68,69,70,71,72,73,74,75,76,77,78,79,80,81,82,83,84,85,86], direct transfer from ambulance to the imaging room [86,87,88,89,90,91,92], strategic location of the imaging room (IR) [93], and routine administration of thrombolysis in the IR, were considered effective strategies to reduce thrombolysis delay [68,69,85,86,87,88,89,94,95]. In places where the physician, usually a neurologist, responsible for thrombolysis, is on-call, pre-notification of possible stroke before imaging also reduced delays [37,68,69,73,84,85,89,90,92,96,97,98,99]. In-hospital use of telestroke is also an option to reduce delays and increase the number of patients receiving thrombolysis [100]. Some studies demonstrated that a protocol containing coordinated actions [62,78,80,87,88], for instance hospital pre-notification by emergency services, direct transfer of patients to the IR immediately after rapid neurological assessment in the emergency room, alteplase preparation soon after first images, rapid interpretation of brain tomography followed by administration of thrombolysis while in the brain CT marked reduced delays and was associated with better outcomes [83,101,102].

### 3.3. Quality Assessment of the Studies

The majority of studies were classified as being of good quality (Appendix A-Table A2). Only three studies were assessed as poor quality. The grey literature search identified 36 articles, none related to the topic of this review.

## 4. Discussion

Under-recognition or delays in recognition of stroke symptoms are consistently identified as one of the main causes of pre-hospital delays [8,16]. Hence, with no surprise, population campaigns focused on stroke recognition, severity and existence of treatment emerged as a common factor to promote ART [8,9,10,11,12,13,14,15,16,17,20,23,34,36,38,39,40,41,46,48,103,104,105,106,107,108]. Sustainable levels of population awareness may only be achieved by continuous education campaigns, probably at least six months each [53]. Evaluation of stroke symptoms by emergency services is not linear and education of pre-hospital professionals also reduced pre-hospital delays [20,22,37,40,51,61,82,99,106,107,109]. Use of ambulance significantly reduced pre and intra-hospital delays, especially if pre-notification is made to the hospital allowing the hospital teams to check the patient’s previous clinical notes and the put everything involved in the chain of care in “preparedness mode” including the availability of the imaging room [12,15,16,17,20,22,23,37,39,40,44,48,51,61,92,96,97,98,110,111,112,113]. For time-dependent treatment, such as the ART, implementation of prehospital [51,61,75,98,107] and intrahospital [48,51,59,67,68,70,71,72,74,76,77,79,84,86,89,90,91,92,108,109,111,114,115,116,117] protocols are fundamental. Although methodology and protocols can be different based on the local resources and geographical conditions, everyone in the chain of care, from the patient in the community, the bystander or witness, to the nurse responsible for delivering alteplase or the neuroradiologist responsible for thrombectomy, should know that each minute after stroke contributes negatively to the prognosis. Intrahospitalar stroke pathways delays should be subject to continuous improvement with implementation of the best available protocols whenever possible. For instance, the “Helsinki model” that includes prehospital with mandatory prenotification, intrahospital with relocation of IR close to the ER admission, direct transfer from ambulance to the IR and delivery of thrombolysis in the IR, reduced dramatically delays in different countries and contexts [83,89,101,102]. Training and inclusion of interventional cardiologists is an alternative solution to improve the availability of endovascular treatment for AIS [118].

Contribution of organizational or management factors must always be contextualized. For instance, in remote areas, protocols for patients with possible stroke should consider the clinical status (severity) and the distance from a center with thrombectomy capability [22,37,56]. This triage would direct patients with a high probability of large vessel occlusion to distant centers with such capability (motherboard model) but only if the patient would arrive within the time window for the treatment. Another example of organizational strategies useful in a specific context is the use of mobile SUs. Past studies show that mobile SUs are cost-effective only when intensive use is anticipated such as in urban areas with high population density [25,27,28,29,30,31,32]. Monitoring of stroke pathways protocols to guarantee consistent performance [76,78] and having the best information available such as the population density and distribution, specific location of the human and material resources [54,76] involved is central for optimizing the chain of stroke care. In order to improve access to ART, priority should be given to the discussion of organizational factors and models of stroke care with the integration of national and regional health facilities. Introduction of advanced imaging techniques in more peripheral regions could lead to more AIS receiving ART. Implementation of mixed prehospital approaches of care, for instance combining mothership with drip-and-ship models for mechanical thrombectomy may be complex, but in some regions would be the sole alternative to increase the number of patients with AIS receiving the best indicated treatment.

## 5. Conclusions

This systematic review identified several organizational factors that determine access to ART. Most of them, for instance population education, promotion of protocols, and training of stroke teams, are mandatory and applicable in any context. There are, however, specific interventions whose application is dependent on the specific population and geographical characteristics.

## Data Availability

Not applicable.

## Appendix A

**Table A1:** List of excluded studies in the systematic literature review.

Reference	Reason for Exclusion
Candelaresi P, Manzo V, Servillo G, Muto M, Barone P, Napoletano R, Saponiero R, Andreone V, Palma V, Spitaleri D, D’Onofrio F, Maniscalco G, Salvatore S, Leone G, Capone E, Schettino C, Romano D, Martusciello G, Miniello S, Mazzaferro MP, Ascione S. The Impact of COVID-19 Lockdown on Stroke Admissions and Treatments in Campania. J Stroke Cerebrovasc Dis. 2021 Jan; 30 (1): 105448. doi: 10.1016/j.jstrokecerebrovasdis.2020.105448. Epub 2020 Nov 4. PMID: 33166767; PMCID: PMC7640890.	Very specific population (COVID-19)
Le Guern V, Rossignol M, Proust A; Comité National d’Experts sur la Mortalité Maternelle. Morts maternelles de causes indirectes (hors AVC, maladies cardiovasculaires et infections) en France 2013–2015 [Indirect causes of maternal deaths (except stroke, cardiovascular diseases and infections) in France 2013-2015]. Gynecol Obstet Fertil Senol. 2021 Jan; 49 (1): 83-88. French. doi: 10.1016/j.gofs.2020.11.015. Epub 2020 Nov 5. PMID: 33161193.	Language (French)
Reddy S, Wu TC, Zhang J, Rahbar MH, Ankrom C, Zha A, Cossey TC, Aertker B, Vahidy F, Parsha K, Jones E, Sharrief A, Savitz SI, Jagolino-Cole A. Lack of Racial, Ethnic, and Sex Disparities in Ischemic Stroke Care Metrics within a Tele-Stroke Network. J Stroke Cerebrovasc Dis. 2021 Jan; 30 (1): 105418. doi: 10.1016/j.jstrokecerebrovasdis.2020.105418. Epub 2020 Nov 2. PMID: 33152594.	No data
Macha K, Hoelter P, Siedler G, Knott M, Schwab S, Doerfler A, Kallmünzer B, Engelhorn T. Multimodal CT or MRI for IV thrombolysis in ischemic stroke with unknown time of onset. Neurology. 2020 Dec 1; 95 (22): e2954-e2964. doi: 10.1212/WNL.0000000000011059. Epub 2020 Oct 21. PMID: 33087492.	No data
von Kleist T, Meyer D, Rapp K, Meyer BC, Modir R. Characteristics and Outcomes of Patients who Refuse Intravenous Thrombolysis for Acute Ischemic Stroke—The San Diego Experience. J Stroke Cerebrovasc Dis. 2020 Nov; 29 (11): 105137. doi: 10.1016/j.jstrokecerebrovasdis.2020.105137. Epub 2020 Aug 4. PMID: 33066942.	Very specific population (patients who refused thrombolysis)
Jaffe TA, Goldstein JN, Yun BJ, Etherton M, Leslie-Mazwi T, Schwamm LH, Zachrison KS. Impact of Emergency Department Crowding on Delays in Acute Stroke Care. West J Emerg Med. 2020 Jul 8; 21 (4): 892-899. doi: 10.5811/westjem.2020.5.45873. PMID: 32726261; PMCID: PMC7390586.	No data
Naccarato M, Scali I, Olivo S, Ajčević M, Buoite Stella A, Furlanis G, Lugnan C, Caruso P, Peratoner A, Cominotto F, Manganotti P. Has COVID-19 played an unexpected “stroke” on the chain of survival? J Neurol Sci. 2020 Jul 15; 414: 116889. doi: 10.1016/j.jns.2020.116889. Epub 2020 May 6. PMID: 32416370; PMCID: PMC7201240	Very specific population (COVID-19)
Krementz NA, Landman A, Gardener HE, Arauz A, Rodriguez AD, Cannon H, Lau HL, Sur N, Marulanda-Londoño E, Yavagal DR, Yan B, Nagel S, Demchuk AM, Khatri P, Romano JG, Asdaghi N. Endovascular Therapy in Mild Ischemic Strokes Presenting Under 6 hours: An International Survey. J Stroke Cerebrovasc Dis. 2020 Nov; 29 (11): 105234. doi: 10.1016/j.jstrokecerebrovasdis.2020.105234. Epub 2020 Aug 20. PMID: 33066890.	Very specific population (minor stroke)
Reiff T, Barthel O, Ringleb PA, Pfaff J, Mundiyanapurath S. Safety of Mechanical Thrombectomy with Combined Intravenous Thrombolysis in Stroke Treatment 4.5 to 9 Hours from Symptom Onset. J Stroke Cerebrovasc Dis. 2020 Nov; 29 (11): 105204. doi: 10.1016/j.jstrokecerebrovasdis.2020.105204. Epub 2020 Aug 13. PMID: 33066886.	Very specific population (Stroke more than 9 hours from onset)
Wang J, Chaudhry SA, Tahsili-Fahadan P, Altaweel LR, Bashir S, Bahiru Z, Fang Y, Qureshi AI. The impact of COVID-19 on acute ischemic stroke admissions: Analysis from a community-based tertiary care center. J Stroke Cerebrovasc Dis. 2020 Dec; 29 (12): 105344. doi: 10.1016/j.jstrokecerebrovasdis.2020.105344. Epub 2020 Sep 25. PMID: 33049464; PMCID: PMC7518171.	Very specific population (COVID 19)
Zini A, Romoli M, Gentile M, Migliaccio L, Picoco C, Dell’Arciprete O, Simonetti L, Naldi F, Piccolo L, Gordini G, Tagliatela F, Bua V, Cirillo L, Princiotta C, Coniglio C, Descovich C, Cortelli P. The stroke mothership model survived during COVID-19 era: an observational single-center study in Emilia-Romagna, Italy. Neurol Sci. 2020 Dec; 41 (12): 3395-3399. doi: 10.1007/s10072-020-04754-2. Epub 2020 Oct 8. PMID: 33030622; PMCID: PMC7541754.	Very specific population (minor stroke/TIA)
John S, Hussain SI, Piechowski-Jozwiak B, Dibu J, Kesav P, Bayrlee A, Elkambergy H, John TLS, Roser F, Mifsud VA. Clinical characteristics and admission patterns of stroke patients during the COVID 19 pandemic: A single center retrospective, observational study from the Abu Dhabi, United Arab Emirates. Clin Neurol Neurosurg. 2020 Dec; 199: 106227. doi: 10.1016/j.clineuro.2020.106227. Epub 2020 Sep 11. PMID: 33011516; PMCID: PMC7485577.	Very specific population (COVID 19)
Kaesmacher J, Maamari B, Meinel TR, Piechowiak EI, Mosimann PJ, Mordasini P, Goeldlin M, Arnold M, Dobrocky T, Boeckh-Behrens T, Berndt M, Michel P, Requena M, Benali A, Pierot L, Mendes Pereira V, Boulouis G, Brehm A, Sporns PB, Ospel JM, Gralla J, Fischer U; BEYOND-SWIFT Investigators. Effect of Pre- and In-Hospital Delay on Reperfusion in Acute Ischemic Stroke Mechanical Thrombectomy. Stroke. 2020 Oct; 51 (10): 2934-2942. doi: 10.1161/STROKEAHA.120.030208. Epub 2020 Sep 16. PMID: 32933420; PMCID: PMC7523579.	No data
Shokri HM, El Nahas NM, Aref HM, Dawood NL, Abushady EM, Abd Eldayem EH, Georgy SS, Zaki AS, Bedros RY, Wahid El Din MM, Roushdy TM. Factors related to time of stroke onset versus time of hospital arrival: A SITS registry-based study in an Egyptian stroke center. PLoS One. 2020 Sep 11; 15 (9): e0238305. doi: 10.1371/journal.pone.0238305. PMID: 32915811; PMCID: PMC7485782.	No data
Qureshi AI, Siddiq F, French BR, Gomez CR, Jani V, Hassan AE, Suri MFK. Effect of COVID-19 Pandemic on Mechanical Thrombectomy for Acute Ischemic Stroke Treatment in United States. J Stroke Cerebrovasc Dis. 2020 Oct; 29 (10): 105140. doi: 10.1016/j.jstrokecerebrovasdis.2020.105140. Epub 2020 Jul 11. PMID: 32912573.	Very specific population (COVID 19)
Xu H, Jia B, Huo X, Mo D, Ma N, Gao F, Yang M, Miao Z. Predictors of Futile Recanalization After Endovascular Treatment in Patients with Acute Ischemic Stroke in a Multicenter Registry Study. J Stroke Cerebrovasc Dis. 2020 Oct; 29 (10): 105067. doi: 10.1016/j.jstrokecerebrovasdis.2020.105067. Epub 2020 Jul 30. PMID: 32912569.	No data
Beckhauser MT, Castro-Afonso LH, Dias FA, Nakiri GS, Monsignore LM, Martins Filho RK, Camilo MR, Aléssio Alves FF, Libardi M, Rodrigues GR, Pontes-Neto OM, Abud DG. Extended Time Window Mechanical Thrombectomy for Acute Stroke in Brazil. J Stroke Cerebrovasc Dis. 2020 Oct; 29 (10): 105134. doi: 10.1016/j.jstrokecerebrovasdis.2020.105134. Epub 2020 Jul 17. PMID: 32912530.	No data
Rao RR, Desai SM, Tonetti DA, Manners J, Gross BA, Jankowitz B, Jovin TG, Jadhav AP. Thrombectomy after in-house stroke in the transfer population. J Stroke Cerebrovasc Dis. 2020 Sep; 29 (9): 105049. doi: 10.1016/j.jstrokecerebrovasdis.2020.105049. Epub 2020 Jun 23. PMID: 32807457.	No data
Mainz J, Andersen G, Valentin JB, Gude MF, Johnsen SP. Disentangling Sex Differences in Use of Reperfusion Therapy in Patients With Acute Ischemic Stroke. Stroke. 2020 Aug; 51 (8): 2332-2338. doi: 10.1161/STROKEAHA.119.028589. Epub 2020 Jul 9. PMID: 32640943.	No data
Hinsenveld WH, de Ridder IR, van Oostenbrugge RJ, van Zwam WH, Vos JA, Coutinho JM, Lycklama À Nijeholt GJ, Boiten J, Schonewille WJ; MR CLEAN Registry Investigators. Intravenous Thrombolysis Is Not Associated with Increased Time to Endovascular Treatment. Cerebrovasc Dis. 2020; 49 (3): 321-327. doi: 10.1159/000508898. Epub 2020 Jul 2. PMID: 32615562.	No data
Desai SM, Tonetti DA, Morrison AA, Molyneaux BJ, Starr M, Rocha M, Gross BA, Jankowitz B, Jovin TG, Jadhav AP. Delayed functional independence after thrombectomy: temporal characteristics and predictors. J Neurointerv Surg. 2020 Sep; 12 (9): 837-841. doi: 10.1136/neurintsurg-2020-016111. Epub 2020 Jun 2. PMID: 32487769.	No data
Yang W, Zhang L, Yao Q, Chen W, Yang W, Zhang S, He L, Li H, Zhang Y. Endovascular treatment or general treatment: how should acute ischemic stroke patients choose to benefit from them the most?: A systematic review and meta-analysis. Medicine (Baltimore). 2020 May; 99 (20): e20187. doi: 10.1097/MD.0000000000020187. PMID: 32443338; PMCID: PMC7254577.	No data
Tiu J, Watson T, Clissold B. Mechanical thrombectomy for emergent large vessel occlusion: an Australian primary stroke centre workflow analysis. Intern Med J. 2020 Apr 7. doi: 10.1111/imj.14843. Epub ahead of print. PMID: 32266746.	No data
Sharobeam A, Jones B, Walton-Sonda D, Lueck CJ. Factors delaying intravenous thrombolytic therapy in acute ischaemic stroke: a systematic review of the literature. J Neurol. 2020 Mar 21. doi: 10.1007/s00415-020-09803-6. Epub ahead of print. PMID: 32206899.	No data
Schlemm L, Endres M, Nolte CH. Bypassing the Closest Stroke Center for Thrombectomy Candidates: What Additional Delay to Thrombolysis Is Acceptable? Stroke. 2020 Mar; 51 (3): 867-875. doi: 10.1161/STROKEAHA.119.027512. Epub 2020 Jan 22. PMID: 31964288.	No data
Adil MM, Luby M, Lynch JK, Hsia AW, Kalaria CP, Nadareishvili Z, Latour LL, Leigh R. Routine use of FLAIR-negative MRI in the treatment of unknown onset stroke. J Stroke Cerebrovasc Dis. 2020 Sep; 29 (9): 105093. doi: 10.1016/j.jstrokecerebrovasdis.2020.105093. Epub 2020 Jul 4. PMID: 32807487	No data
Kamal N, Jeerakathil T, Stang J, Liu M, Rogers E, Smith EE, Demchuk AM, Siddiqui M, Mann B, Bestard J, Lang E, Shand E, Benard M, Collins L, Martin K, Hartley C, Reiber M, Valaire S, Mrklas KJ, Hill MD; QuICR Alberta Stroke Program. Provincial Door-to-Needle Improvement Initiative Results in Improved Patient Outcomes Across an Entire Population. Stroke. 2020 Aug; 51 (8): 2339-2346. doi: 10.1161/STROKEAHA.120.029734. Epub 2020 Jul 9. PMID: 32640947.	No data
Gardiner FW, Bishop L, Dos Santos A, Sharma P, Easton D, Quinlan F, Churilov L, Schwarz M, Walter S, Fassbender K, Davis SM, Donnan GA. Aeromedical Retrieval for Stroke in Australia. Cerebrovasc Dis. 2020; 49 (3): 334-340. doi: 10.1159/000508578. Epub 2020 Jun 24. PMID: 32580203	No data
Abe A, Ota T, Ueda M, Amano T, Shigeta K, Matsumaru Y, Shiokawa Y, Hirano T. Tokyo Metropolitan Stroke Emergency Medical Services for Interventional Stroke Treatment: The Tama-REgistry of Acute Thrombectomy (TREAT) Study. J Stroke Cerebrovasc Dis. 2020 Jun; 29 (6): 104752. doi: 10.1016/j.jstrokecerebrovasdis.2020.104752. Epub 2020 Apr 7. PMID: 32276861.	No data
Watson T, Tiu J, Clissold B. Addressing inequity in acute stroke care requires attention to each component of regional workflow. Med J Aust. 2020 Jan; 212 (1): 8-10.e1. doi: 10.5694/mja2.50440. Epub 2019 Nov 29. PMID: 31785010.	No data
Colton K, Richards CT, Pruitt PB, Mendelson SJ, Holl JL, Naidech AM, Prabhakaran S, Maas MB. Early Stroke Recognition and Time-based Emergency Care Performance Metrics for Intracerebral Hemorrhage. J Stroke Cerebrovasc Dis. 2020 Feb; 29 (2): 104552. doi: 10.1016/j.jstrokecerebrovasdis.2019.104552. Epub 2019 Dec 12. PMID: 31839545; PMCID: PMC6954314.	No data
Wilhelm LO, Gellert P, White M, Araujo-Soares V, Ford GA, Mackintosh JE, Rodgers H, Sniehotta FF, Thomson RG, Dombrowski SU. The Recognition-Response Gap in Acute Stroke: Examining the Relationship between Stroke Recognition and Response in a General Population Survey. J Stroke Cerebrovasc Dis. 2020 Feb; 29 (2): 104499. doi: 10.1016/j.jstrokecerebrovasdis.2019.104499. Epub 2019 Nov 19. PMID: 31757598.	No data
Borghese O, Pisani A, Lapergue B, Di Centa I. Early Carotid Endarterectomy for Symptomatic Stenosis of Internal Carotid Artery in Patients Affected by Transient Ischemic Attack or Minor-to-Moderate Ischemic Acute Stroke: A Single-Center Experience. Ann Vasc Surg. 2020 May; 65: 232-239. doi: 10.1016/j.avsg.2019.10.088. Epub 2019 Nov 6. PMID: 31705984.	No data
Gennesseaux J, Giordano Orsini G, Lefour S, Bakchine S, Marion Q, Barbe C, Gennai S. Early Management of Transient Ischemic Attack in Emergency Departments in France. J Stroke Cerebrovasc Dis. 2020 Jan; 29 (1): 104464. doi: 10.1016/j.jstrokecerebrovasdis.2019.104464. Epub 2019 Nov 4. PMID: 31699576.	No data
Alonso de Leciñana M, Mazya MV, Kostulas N, Del Brutto OH, Abanto C, Massaro AR, de Bastos M, Martins S, Ameriso SF, Gongora-Rivera F, Sacks C, Hoppe A, Abad P, Meza G, Arauz-Gongora A, Wahlgren N, Díez-Tejedor E; SITS-SIECV Investigators. Stroke Care and Application of Thrombolysis in Ibero-America: Report From the SITS-SIECV Ibero-American Stroke Register. Stroke. 2019 Sep; 50 (9): 2507–2512. doi: 10.1161/STROKEAHA.119.025668. Epub 2019 Aug 6. PMID: 31670921.	No data
Iglesias Mohedano AM, García Pastor A, de Lorenzo Pinto A, Gil Núñez AC. Bolus-to-Infusion Time in Stroke Patients Treated with Alteplase on the CT Table: A Clinical Concern. Cerebrovasc Dis. 2019; 48 (1-2): 96-98. doi: 10.1159/000503338. Epub 2019 Oct 8. PMID: 31593952.	No data
Lima FO, Mont’Alverne FJA, Bandeira D, Nogueira RG. Pre-hospital Assessment of Large Vessel Occlusion Strokes: Implications for Modeling and Planning Stroke Systems of Care. Front Neurol. 2019 Sep 13; 10: 955. doi: 10.3389/fneur.2019.00955. PMID: 31572286; PMCID: PMC6753197.	No data
Adams NC, Griffin E, Motyer R, Farrell T, Carmody E, O’Shea A, Murphy B, O’Hare A, Looby S, Power S, Brennan P, Doyle KM, Thornton J. Review of external referrals to a regional stroke centre: it is not just about thrombectomy. Clin Radiol. 2019 Dec; 74 (12): 950-955. doi: 10.1016/j.crad.2019.07.021. Epub 2019 Sep 11. PMID: 31521325.	No data
Fekadu G, Adola B, Mosisa G, Shibiru T, Chelkeba L. Clinical characteristics and treatment outcomes among stroke patients hospitalized to Nekemte referral hospital, western Ethiopia. J Clin Neurosci. 2020 Jan; 71: 170-176. doi: 10.1016/j.jocn.2019.08.075. Epub 2019 Aug 27. PMID: 31471079.	No data
El Nahas NM, Shokri HM, Roushdy TM, Aref HM, Hamed SM, Shalash AS, Abdelrahem MI, Dawood NL, Abushady EM, Georgy SS, Zaki AS, Bedros RY. Urban Versus Rural Egypt: Stroke Risk Factors and Clinical Profile: Cross-Sectional Observational Study. J Stroke Cerebrovasc Dis. 2019 Nov; 28 (11): 104316. doi: 10.1016/j.jstrokecerebrovasdis.2019.104316. Epub 2019 Aug 12. PMID: 31416762	No data
Tanaka K, Matsumoto S, Yamada T, Nagano S, Takase KI, Hatano T, Yamasaki R, Kira JI. Temporal Trends in Clinical Characteristics and Door-to-Needle Time in Patients Receiving Intravenous Tissue Plasminogen Activator: A Retrospective Study of 4 Hospitals in Japan. J Stroke Cerebrovasc Dis. 2019 Nov; 28 (11): 104305. doi: 10.1016/j.jstrokecerebrovasdis.2019.104305. Epub 2019 Aug 10. PMID: 31405791.	No data
Ueno T, Nishijima H, Hikichi H, Haga R, Arai A, Suzuki C, Nunomura JI, Saito K, Tomiyama M. Helicopter Transport for Patients with Cerebral Infarction in Rural Japan. J Stroke Cerebrovasc Dis. 2019 Sep; 28 (9): 2525-2529. doi: 10.1016/j.jstrokecerebrovasdis.2019.06.010. Epub 2019 Jun 27. PMID: 31256983.	No data
Morii Y, Osanai T, Ishikawa T, Fujiwara K, Tanikawa T, Houkin K, Kobayashi E, Ogasawara K. Cost Effectiveness of Drive and Retrieve System in Hokkaido for Acute Ischemic Stroke Patient Treatment Using Geographic Information System. J Stroke Cerebrovasc Dis. 2019 Aug; 28 (8): 2292-2301. doi: 10.1016/j.jstrokecerebrovasdis.2019.05.020. Epub 2019 Jun 12. PMID: 31200963.	No data
Greenberg K, Hedayat HS, Binning MJ, Veznedaroglu E. Innovations in Care Delivery of Stroke from Emergency Medical Services to the Neurointerventional Operating Room. Neurosurgery. 2019 Jul 1; 85 (suppl_1): S18-S22. doi: 10.1093/neuros/nyz021. PMID: 31197327.	No data
Niklasson A, Herlitz J, Jood K. Socioeconomic disparities in prehospital stroke care. Scand J Trauma Resusc Emerg Med. 2019 May 2; 27 (1): 53. doi: 10.1186/s13049-019-0630-6. PMID: 31046804; PMCID: PMC6498576.	No data
Park J, Choi KH, Lee JM, Kim HK, Hwang D, Rhee TM, Kim J, Park TK, Yang JH, Song YB, Choi JH, Hahn JY, Choi SH, Koo BK, Chae SC, Cho MC, Kim CJ, Kim JH, Jeong MH, Gwon HC, Kim HS; KAMIR-NIH (Korea Acute Myocardial Infarction Registry–National Institutes of Health) Investigators. Prognostic Implications of Door-to-Balloon Time and Onset-to-Door Time on Mortality in Patients With ST -Segment-Elevation Myocardial Infarction Treated With Primary Percutaneous Coronary Intervention. J Am Heart Assoc. 2019 May 7; 8 (9): e012188. doi: 10.1161/JAHA.119.012188. PMID: 31041869; PMCID: PMC6512115.	No data
Lu MY, Chen CH, Yeh SJ, Tsai LK, Lee CW, Tang SC, Jeng JS. Comparison between in-hospital stroke and community-onset stroke treated with endovascular thrombectomy. PLoS One. 2019 Apr 12; 14 (4): e0214883. doi: 10.1371/journal.pone.0214883. PMID: 30978233; PMCID: PMC6461247.	No data
Trent SA, Morse EA, Ginde AA, Havranek EP, Haukoos JS. Barriers to Prompt Presentation to Emergency Departments in Colorado after Onset of Stroke Symptoms. West J Emerg Med. 2019 Mar; 20 (2): 237-243. doi: 10.5811/westjem.2018.10.38731. Epub 2018 Dec 5. PMID: 30881542; PMCID: PMC6404721.	No data
Venema E, Lingsma HF, Chalos V, Mulder MJHL, Lahr MMH, van der Lugt A, van Es ACGM, Steyerberg EW, Hunink MGM, Dippel DWJ, Roozenbeek B. Personalized Prehospital Triage in Acute Ischemic Stroke. Stroke. 2019 Feb; 50 (2): 313-320. doi: 10.1161/STROKEAHA.118.022562. PMID: 30661502; PMCID: PMC6358183.	No data (Sample of Convenience)
Huang Q, Song HQ, Ma QF, Song XW, Wu J. Effects of time delays on the therapeutic outcomes of intravenous thrombolysis for acute ischemic stroke in the posterior circulation: An observational study. Brain Behav. 2019 Feb; 9 (2): e01189. doi: 10.1002/brb3.1189. Epub 2019 Jan 6. PMID: 30614220; PMCID: PMC6379513.	No data
Zhang C, Lou M, Chen Z, Chen H, Xu D, Wang Z, Hu H, Wu C, Zhang X, Ma X, Wang Y, Hu H. [Analysis of intravenous thrombolysis time and prognosis in patients with in-hospital stroke]. Zhejiang Da Xue Xue Bao Yi Xue Ban. 2019 May 25; 48 (3): 260-266. Chinese. PMID: 31496157	Language (Chinese)
Leibinger F, Sablot D, Van Damme L, Gaillard N, Nguyen Them L, Lachcar M, Duchateau N, Arquizan C, Farouil G, Ibanez M, Pujol C, Fadat B, Allou T, Coll F, Benayoun L, Mas J, Smadja P, Ferraro-Allou A, Mourand I, Dutray A, Tardieu M, Jurici S, Bonnec JM, Olivier N, Cardini S, Aptel S, Marquez AM, Dumitrana A, Costalat V, Bonafe A. Which Patients Require Physician-Led Inter-Hospital Transport in View of Endovascular Therapy? Cerebrovasc Dis. 2019; 48 (3-6): 171-178. doi: 10.1159/000504314. Epub 2019 Nov 14. PMID: 31726450.	No data
Dhand A, Luke D, Lang C, Tsiaklides M, Feske S, Lee JM. Social networks and risk of delayed hospital arrival after acute stroke. Nat Commun. 2019 Mar 14; 10 (1): 1206. doi: 10.1038/s41467-019-09073-5. PMID: 30872570; PMCID: PMC6418151.	No data
Muñío-Iranzo ML, Marta-Enguita J, Marta-Moreno J, Gasch-Gallén A, Sampériz-Murillo M. Casuística de códigos ictus atendidos por 061 ARAGÓN en el período 2010-2016. Factores que influyen en los tiempos de respuesta y de acceso a la fibrinólisis [Characteristics of the stroke alert process attended by 061 ARAGON assistance units from 2010 to 2016. Factors influencing times of response and access to fibrinolytic treatment]. Rev Neurol. 2019 Nov 16; 69 (10): 409-416. Spanish. doi: 10.33588/rn.6910.2019117. PMID: 31713227.	No data
George BP, Pieters TA, Zammit CG, Kelly AG, Sheth KN, Bhalla T. Trends in Interhospital Transfers and Mechanical Thrombectomy for United States Acute Ischemic Stroke Inpatients. J Stroke Cerebrovasc Dis. 2019 Apr; 28 (4): 980-987. doi: 10.1016/j.jstrokecerebrovasdis.2018.12.018. Epub 2019 Jan 8. PMID: 30630752.	No data
Vaughan Sarrazin M, Limaye K, Samaniego EA, Al Kasab S, Sheharyar A, Dandapat S, Guerrero WR, Hasan DM, Ortega-Gutierrez S, Derdeyn CP, Torner JC, Chamorro A, Leira EC. Disparities in Inter-hospital Helicopter Transportation for Hispanics by Geographic Region: A Threat to Fairness in the Era of Thrombectomy. J Stroke Cerebrovasc Dis. 2019 Mar; 28 (3): 550-556. doi: 10.1016/j.jstrokecerebrovasdis.2018.10.031. Epub 2018 Dec 11. PMID: 30552028.	No data
Pham H, Russell T, Seiwert A, Kasper G, Lurie F. Timing of Hospital-acquired Venous Thromboembolism and Its Relationship with Venous Thromboembolism Prevention Measures in Immobile Patients. Ann Vasc Surg. 2019 Apr; 56: 24-28. doi: 10.1016/j.avsg.2018.09.014. Epub 2018 Nov 27. PMID: 30500652.	No data
Mackay MH, Ratner PA, Veenstra G, Scheuermeyer FX, Grubisic M, Ramanathan K, Murray C, Humphries KH. Racism Is Not a Factor in Door-to-electrocardiogram Times of Patients With Symptoms of Acute Coronary Syndrome: A Prospective, Observational Study. Acad Emerg Med. 2019 May; 26 (5): 491-500. doi: 10.1111/acem.13569. Epub 2018 Oct 21. PMID: 30222233; PMCID: PMC6563064.	No data
Allen M, Pearn K, Monks T, Bray BD, Everson R, Salmon A, James M, Stein K. Can clinical audits be enhanced by pathway simulation and machine learning? An example from the acute stroke pathway. BMJ Open. 2019 Sep 17; 9 (9): e028296. doi: 10.1136/bmjopen-2018-028296. PMID: 31530590; PMCID: PMC6756466.	No data
Adams KM, Burns PA, Hunter A, Rennie I, Flynn PA, Smyth G, Gordon PL, Patterson CE, Fearon P, Kerr ELF, Wiggam MI. Outcomes after Thrombectomy in Belfast: Mothership and Drip-and-Ship in the Real World. Cerebrovasc Dis. 2019; 47 (5-6): 231-237. doi: 10.1159/000500849. Epub 2019 Jun 18. PMID: 31212294	No data
Javor A, Ferrari J, Posekany A, Asenbaum-Nan S. Stroke risk factors and treatment variables in rural and urban Austria: An analysis of the Austrian Stroke Unit Registry. PLoS One. 2019 Apr 10; 14 (4): e0214980. doi: 10.1371/journal.pone.0214980. PMID: 30970026; PMCID: PMC6457636	No data
Hayes M, Schlundt D, Bonnet K, Vogus TJ, Kripalani S, Froehler MT, Ward MJ. Tales from the Trips: A Qualitative Study of Timely Recognition, Treatment, and Transfer of Emergency Department Patients with Acute Ischemic Stroke. J Stroke Cerebrovasc Dis. 2019 May; 28 (5): 1219-1228. doi: 10.1016/j.jstrokecerebrovasdis.2019.01.012. Epub 2019 Feb 7. PMID: 30745000; PMCID: PMC6467718.	No data
Goyal M, Almekhlafi M, Dippel DW, Campbell BCV, Muir K, Demchuk AM, Bracard S, Davalos A, Guillemin F, Jovin TG, Menon BK, Mitchell PJ, Brown S, White P, Majoie CBLM, Saver JL, Hill MD; HERMES Collaborators. Rapid Alteplase Administration Improves Functional Outcomes in Patients With Stroke due to Large Vessel Occlusions. Stroke. 2019 Mar; 50 (3): 645-651. doi: 10.1161/STROKEAHA.118.021840. PMID: 30760169.	No data
Bourcier R, Goyal M, Liebeskind DS, Muir KW, Desal H, Siddiqui AH, Dippel DWJ, Majoie CB, van Zwam WH, Jovin TG, Levy EI, Mitchell PJ, Berkhemer OA, Davis SM, Derraz I, Donnan GA, Demchuk AM, van Oostenbrugge RJ, Kelly M, Roos YB, Jahan R, van der Lugt A, Sprengers M, Velasco S, Lycklama À Nijeholt GJ, Ben Hassen W, Burns P, Brown S, Chabert E, Krings T, Choe H, Weimar C, Campbell BCV, Ford GA, Ribo M, White P, Cloud GC, San Roman L, Davalos A, Naggara O, Hill MD, Bracard S; HERMES Trialists Collaboration. Association of Time From Stroke Onset to Groin Puncture With Quality of Reperfusion After Mechanical Thrombectomy: A Meta-analysis of Individual Patient Data From 7 Randomized Clinical Trials. JAMA Neurol. 2019 Apr 1; 76 (4): 405-411. doi: 10.1001/jamaneurol.2018.4510. Erratum in: JAMA Neurol. 2019 May 28; PMID: 30667465; PMCID: PMC6459219.	No data
Hoepner R, Weber R, Reimann G, Berger K, Kitzrow M, Fischer S, Weimar C, Eyding J, Krogias C. Stroke admission outside daytime working hours delays mechanical thrombectomy and worsens short-term outcome. Int J Stroke. 2019 Jul; 14 (5): 517-521. doi: 10.1177/1747493018790079. Epub 2018 Jul 19. PMID: 30024313.	No data
Bandettini di Poggio M, Finocchi C, Brizzo F, Altomonte F, Bovis F, Mavilio N, Serrati C, Malfatto L, Mancardi G, Balestrino M. Management of acute ischemic stroke, thrombolysis rate, and predictors of clinical outcome. Neurol Sci. 2019 Feb; 40 (2): 319-326. doi: 10.1007/s10072-018-3644-3. Epub 2018 Nov 14. PMID: 30430315	No data
Ali SF, Fonarow G, Liang L, Xian Y, Smith EE, Bhatt DL, Schwamm L. Rates, Characteristics, and Outcomes of Patients Transferred to Specialized Stroke Centers for Advanced Care. Circ Cardiovasc Qual Outcomes. 2018 Sep; 11 (9): e003359. doi: 10.1161/CIRCOUTCOMES.116.003359. PMID: 30354551	No data
Alberts MJ, Ollenschleger MD, Nouh A. Dawn of a New Era for Stroke Treatment: Implications of the DAWN Study for Acute Stroke Care and Stroke Systems of Care. Circulation. 2018 Apr 24; 137 (17): 1767-1769. doi: 10.1161/CIRCULATIONAHA.118.033579. Epub 2018 Jan 18. PMID: 29348262	No data
McTaggart RA, Moldovan K, Oliver LA, Dibiasio EL, Baird GL, Hemendinger ML, Haas RA, Goyal M, Wang TY, Jayaraman MV. Door-in-Door- Out Time at Primary Stroke Centers May Predict Outcome for Emergent Large Vessel Occlusion Patient. Stroke. 2018 Dec; 49 (12): 2969-2974. doi: 10.1161/STROKEAHA.118.021936. PMID: 30571428.	No data
Requena M, Pérez de la Ossa N, Abilleira S, Cardona P, Urra X, Martí-Fabregas J, Rodríguez-Campello A, Boned S, Rubiera M, Tomasello A, Molina CA, Ribo M; for Catalan Stroke Code and Reperfusion Consortium. Predictors of Endovascular Treatment Among Stroke Codes Activated Within 6 Hours From Symptom Onset. Stroke. 2018 Sep; 49 (9): 2116-2121. doi: 10.1161/STROKEAHA.118.021316. PMID: 30354973.	No data
Kuhrij LS, Wouters MW, van den Berg-Vos RM, de Leeuw FE, Nederkoorn PJ. The Dutch Acute Stroke Audit: Benchmarking acute stroke care in the Netherlands. Eur Stroke J. 2018 Dec; 3 (4): 361-368. doi: 10.1177/2396987318787695. Epub 2018 Jul 11. PMID: 31236484; PMCID: PMC6571504.	No data
Koizumi S, Ota T, Shigeta K, Amano T, Ueda M, Matsumaru Y, Shiokawa Y, Hirano T. Onset to Reperfusion Time Was Not Important in Mechanical Thrombectomy for Elderly Patients: A Retrospective Multicenter Study in Tama Area, Tokyo. Cerebrovasc Dis. 2018; 46 (1-2): 89-96. doi: 10.1159/000492867. Epub 2018 Sep 6. PMID: 30189424.	No data
Ota T, Shigeta K, Amano T, Ueda M, Hirano T, Matsumaru Y, Shiokawa Y. Regionwide Retrospective Survey of Acute Mechanical Thrombectomy in Tama, Suburban Tokyo: A Preliminary Report. J Stroke Cerebrovasc Dis. 2018 Nov; 27 (11): 3350-3355. doi: 10.1016/j.jstrokecerebrovasdis.2018.07.044. Epub 2018 Aug 25. PMID: 30154049.	No data
Demaerschalk BM, Boyd EL, Barrett KM, Gamble DM, Sonchik S, Comer MM, Wieser J, Hentz JG J, Fitz-Patrick D, Chang YH. Comparison of Stroke Outcomes of Hub and Spoke Hospital Treated Patients in Mayo Clinic Telestroke Program. J Stroke Cerebrovasc Dis. 2018 Nov; 27 (11): 2940-2942. doi: 10.1016/j.jstrokecerebrovasdis.2018.06.024. Epub 2018 Aug 23. PMID: 30146388.	No data
Faine BA, Dayal S, Kumar R, Lentz SR, Leira EC. Helicopter “Drip and Ship” Flights Do Not Alter the Pharmacological Integrity of rtPA. J Stroke Cerebrovasc Dis. 2018 Oct; 27 (10): 2720-2724. doi: 10.1016/j.jstrokecerebrovasdis.2018.05.049. Epub 2018 Jul 20. PMID: 30037651; PMCID: PMC6139266.	No data
Pressler H, Reich A, Schulz JB, Nikoubashman O, Willmes K, Habib P, Bach JP. Modern Interdisciplinary and Interhospital Acute Stroke Therapy-What Patients Think About It and What They Really Understand. J Stroke Cerebrovasc Dis. 2018 Oct; 27 (10): 2669-2676. doi: 10.1016/j.jstrokecerebrovasdis.2018.05.029. Epub 2018 Jun 30. PMID: 29970323.	No data
Henderson SJ, Weitz JI, Kim PY. Fibrinolysis: strategies to enhance the treatment of acute ischemic stroke. J Thromb Haemost. 2018 Oct; 16 (10): 1932-1940. doi: 10.1111/jth.14215. Epub 2018 Jul 20. PMID: 29953716.	No data
Huang Q, Zhang JZ, Xu WD, Wu J. Generalization of the right acute stroke promotive strategies in reducing delays of intravenous thrombolysis for acute ischemic stroke: A meta-analysis. Medicine (Baltimore). 2018 Jun; 97 (25): e11205. doi: 10.1097/MD.0000000000011205. PMID: 29924046; PMCID: PMC6024468.	No data
Lachkhem Y, Rican S, Minvielle É. Understanding delays in acute stroke care: a systematic review of reviews. Eur J Public Health. 2018 Jun 1; 28 (3): 426-433. doi: 10.1093/eurpub/cky066. PMID: 29790991.	No data
Regenhardt RW, Mecca AP, Flavin SA, Boulouis G, Lauer A, Zachrison KS, Boomhower J, Patel AB, Hirsch JA, Schwamm LH, Leslie-Mazwi TM. Delays in the Air or Ground Transfer of Patients for Endovascular Thrombectomy. Stroke. 2018 Jun; 49 (6): 1419-1425. doi: 10.1161/STROKEAHA.118.020618. Epub 2018 Apr 30. PMID: 29712881; PMCID: PMC5970980.	No data
Zhao H, Pesavento L, Coote S, Rodrigues E, Salvaris P, Smith K, Bernard S, Stephenson M, Churilov L, Yassi N, Davis SM, Campbell BCV. Ambulance Clinical Triage for Acute Stroke Treatment: ParamedicTriage Algorithm for Large Vessel Occlusion. Stroke. 2018 Apr; 49 (4): 945-951. doi: 10.1161/STROKEAHA.117.019307. Epub 2018 Mar 14. PMID: 29540611.	No data
Iglesias Mohedano AM, García Pastor A, Díaz Otero F, Vázquez Alen P, Martín Gómez MA, Simón Campo P, Salgado Cámara P, Esteban de Antonio E, Lázaro García E, Funes Molina C, Del Valle Diéguez M, Saura Lorente J, Fernández Bullido Y, Gil Nuñez A. A new protocol reduces median door-to-needle time to the benchmark of 30 minutes in acute stroke treatment. Neurologia. 2018 Jun 14: S0213-4853 (18)30119-1. English, Spanish. doi: 10.1016/j.nrl.2018.04.001. Epub ahead of print. PMID: 29910083.	No data
Farrag MA, Oraby MI, Ghali AA, Ragab OA, Nasreldein A, Shehata GA, Elfar E, Abd-Allah F. Public stroke knowledge, awareness, and response to acute stroke: Multi-center study from 4 Egyptian governorates. J Neurol Sci. 2018 Jan 15; 384: 46-49. doi: 10.1016/j.jns.2017.11.003. Epub 2017 Nov 4. PMID: 29249376.	No data
Kamal N, Smith EE, Jeerakathil T, Hill MD. Thrombolysis: Improving door-to-needle times for ischemic stroke treatment—A narrative review. Int J Stroke. 2018 Apr; 13 (3): 268-276. doi: 10.1177/1747493017743060. Epub 2017 Nov 15. PMID: 29140185.	No data
Toudou Daouda M, Bouchal S, Chtaou N, Midaoui A, Souirti Z, Belahsen F.Thrombolysis Alert in Hassan II University Teaching Hospital of Fez (Morocco): A Prospective Study of 2 Years J Stroke Cerebrovasc Dis. 2018 Apr; 27 (4): 1100-1106. doi: 10.1016/j.jstrokecerebrovasdis.2017.11.022. Epub 2017 Dec 29. PMID: 29290532.	No data
English SW, Rabinstein AA, Mandrekar J, Klaas JP. Rethinking Prehospital Stroke Notification: Assessing Utility of Emergency Medical Services Impression and Cincinnati Prehospital Stroke Scale. J Stroke Cerebrovasc Dis. 2018 Apr; 27 (4): 919-925. doi: 10.1016/j.jstrokecerebrovasdis.2017.10.036. Epub 2017 Dec 6. PMID: 29217362.	No data
Fujiwara K, Osanai T, Kobayashi E, Tanikawa T, Kazumata K, Tokairin K, Houkin K, Ogasawara K. Accessibility to Tertiary Stroke Centers in Hokkaido, Japan: Use of Novel Metrics to Assess Acute Stroke Care Quality. J Stroke Cerebrovasc Dis. 2018 Jan; 27 (1): 177-184. doi: 10.1016/j.jstrokecerebrovasdis.2017.08.013. Epub 2017 Sep 11. PMID: 28911996.	No data
Venema E, Boodt N, Berkhemer OA, Rood PPM, van Zwam WH, van Oostenbrugge RJ, van der Lugt A, Roos YBWEM, Majoie CBLM, Lingsma HF, Dippel DWJ; MR CLEAN investigators. Workflow and factors associated with delay in the delivery of intra-arterial treatment for acute ischemic stroke in the MR CLEAN trial. J Neurointerv Surg. 2018 May; 10 (5): 424-428. doi: 10.1136/neurintsurg-2017-013198. Epub 2017 Aug 30. PMID: 28855345.	No data
Alawieh A, Pierce AK, Vargas J, Turk AS, Turner RD, Chaudry MI, Spiotta AM. The golden 35 min of stroke intervention with ADAPT: effect of thrombectomy procedural time in acute ischemic stroke on outcome. J Neurointerv Surg. 2018 Mar; 10 (3): 213-220. doi: 10.1136/neurintsurg-2017-013040. Epub 2017 May 2. PMID: 28465405.	No data
García Ruiz R, Silva Fernández J, García Ruiz RM, Recio Bermejo M, Arias Arias Á, Santos Pinto A, Lomas Meneses A, Botía Paniagua E, Abellán Alemán J. Factors related to immediate response to symptoms in patients with stroke or transient ischaemic attack. Neurologia. 2020 Oct; 35 (8): 551-555. English, Spanish. doi: 10.1016/j.nrl.2017.09.013. Epub 2017 Dec 24. PMID: 29279254.	No data
Pulvers JN, Watson JDG. If Time Is Brain Where Is the Improvement in Prehospital Time after Stroke? Front Neurol. 2017 Nov 20; 8: 617. doi: 10.3389/fneur.2017.00617. PMID: 29209269; PMCID: PMC5701972.	No data
Madsen TE, Roberts ET, Kuczynski H, Goldmann E, Parikh NS, Boden-Albala B. Gender, Social Networks, and Stroke Preparedness in the Stroke Warning Information and Faster Treatment Study. J Stroke Cerebrovasc Dis. 2017 Dec; 26 (12): 2734-2741. doi: 10.1016/j.jstrokecerebrovasdis.2017.06.046. Epub 2017 Aug 12. PMID: 28807486.	No data
Seno S, Tomura S, Ono K, Akitomi S, Sekine Y, Yoshimura Y, Tanaka Y, Ikeuchi H, Saitoh D. The Relationship between Functional Outcome and Prehospital Time Interval in Patients with Cerebral Infarction. J Stroke Cerebrovasc Dis. 2017 Dec; 26 (12): 2800-2805. doi: 10.1016/j.jstrokecerebrovasdis.2017.06.059. Epub 2017 Jul 31. PMID: 28774793.	No data
Oluwole SA, Wang K, Dong C, Ciliberti-Vargas MA, Gutierrez CM, Yi L, Romano JG, Perez E, Tyson BA, Ayodele M, Asdaghi N, Gardener H, Rose DZ, Garcia EJ, Zevallos JC, Foster D, Robichaux M, Waddy SP, Sacco RL, Rundek T; FL-PR Collaboration to Reduce Stroke Disparities Investigators. Disparities and Trends in Door-to-Needle Time: The FL-PR CReSD Study (Florida-Puerto Rico Collaboration to Reduce Stroke Disparities). Stroke. 2017 Aug; 48 (8): 2192-2197. doi: 10.1161/STROKEAHA.116.016183. Epub 2017 Jul 13. PMID: 28706119; PMCID: PMC5639478.	No data
Zweifler RM. Initial Assessment and Triage of the Stroke Patient. Prog Cardiovasc Dis. 2017 May-Jun; 59 (6): 527-533. doi: 10.1016/j.pcad.2017.04.004. Epub 2017 Apr 27. PMID: 28457790.	No data
Kelly KM, Holt KT, Neshewat GM, Skolarus LE. Community Interventions to Increase Stroke Preparedness and Acute Stroke Treatment Rates. Curr Atheroscler Rep. 2017 Nov 16; 19 (12): 64. doi: 10.1007/s11883-017-0695-5. PMID: 29147858.	No data
Ishihara H, Oka F, Oku T, Shinoyama M, Suehiro E, Sugimoto K, Suzuki M. Safety and Time Course of Drip-and-Ship in Treatment of Acute Ischemic Stroke. J Stroke Cerebrovasc Dis. 2017 Nov; 26 (11): 2477-2481. doi: 10.1016/j.jstrokecerebrovasdis.2017.03.008. Epub 2017 Sep 19. PMID: 28935501.	No data
Kalnins A, Mickelsen LJ, Marsh D, Zorich C, Casal S, Tai WA, Vora N, Olalia G, Wintermark M, Larson DB. Decreasing Stroke Code to CT Time in Patients Presenting with Stroke Symptoms. Radiographics. 2017 Sep-Oct; 37 (5): 1559-1568. doi: 10.1148/rg.2017160190. Epub 2017 Aug 18. PMID: 28820652.	No data
Chompoopong P, Rostambeigi N, Kassar D, Maud A, Qureshi IA, Cruz-Flores S, Rodriguez GJ. Are We Overlooking Stroke Chameleons? A Retrospective Study on the Delayed Recognition of Stroke Patients. Cerebrovasc Dis. 2017; 44 (1-2): 83-87. doi: 10.1159/000471929. Epub 2017 May 17. PMID: 28511184.	No data
Jewett L, Mirian A, Connolly B, Silver FL, Sahlas DJ. Use of Geospatial Modeling to Evaluate the Impact of Telestroke on Access to Stroke Thrombolysis in Ontario. J Stroke Cerebrovasc Dis. 2017 Jul; 26 (7): 1400-1406. doi: 10.1016/j.jstrokecerebrovasdis.2017.03.023. Epub 2017 May 3. PMID: 28478980.	No data
Zweifler RM. Initial Assessment and Triage of the Stroke Patient. Prog Cardiovasc Dis. 2017 May-Jun; 59 (6): 527-533. doi: 10.1016/j.pcad.2017.04.004. Epub 2017 Apr 27. PMID: 28457790.	No data
Meretoja A, Keshtkaran M, Tatlisumak T, Donnan GA, Churilov L. Endovascular therapy for ischemic stroke: Save a minute-save a week. Neurology. 2017 May 30; 88 (22): 2123-2127. doi: 10.1212/WNL.0000000000003981. Epub 2017 Apr 28. PMID: 28455382.	No data
Sauser Zachrison K, Levine DA, Fonarow GC, Bhatt DL, Cox M, Schulte P, Smith EE, Suter RE, Xian Y, Schwamm LH. Timely Reperfusion in Stroke and Myocardial Infarction Is Not Correlated: An Opportunity for Better Coordination of Acute Care. Circ Cardiovasc Qual Outcomes. 2017 Mar; 10 (3): e003148. doi: 10.1161/CIRCOUTCOMES.116.003148. PMID: 28283469; PMCID: PMC5369604.	No data
Angamuthu N, Queck KK, Menon S, Ho SS, Ang E, De Silva DA. Surveys of Stroke Patients and Their Next of Kin on Their Opinions towards Decision-Making and Consent for Stroke Thrombolysis. Ann Acad Med Singap. 2017 Feb; 46 (2): 50-63. PMID: 28263342.	No data
Gandolfi M, Geroin C, Tomelleri C, Maddalena I, Kirilova Dimitrova E, Picelli A, Smania N, Waldner A. Feasibility and safety of early lower limb robot-assisted training in sub-acute stroke patients: a pilot study. Eur J Phys Rehabil Med. 2017 Dec; 53 (6): 870-882. doi: 10.23736/S1973-9087.17.04468-9. Epub 2017 Jan 12. PMID: 28084064.	No data
Guzauskas GF, Chen E, Lalla D, Yu E, Tayama D, Veenstra DL. What is the value of conducting a trial of r-tPA for the treatment of mild stroke patients? Int J Stroke. 2017 Feb; 12 (2): 137-144. doi: 10.1177/1747493016669887. Epub 2016 Oct 10. PMID: 28134053.	No data
Giray S, Ozdemir O, Baş DF, İnanç Y, Arlıer Z, Kocaturk O. Does stroke etiology play a role in predicting outcome of acute stroke patients who underwent endovascular treatment with stent retrievers? J Neurol Sci. 2017 Jan 15; 372: 104-109. doi: 10.1016/j.jns.2016.11.006. Epub 2016 Nov 10. PMID: 28017193.	No data
Venketasubramanian N, Lee CF, Young SH, Tay SS, Umapathi T, Lao AY, Gan HH, Baroque Ii AC, Navarro JC, Chang HM, Advincula JM, Muengtaweepongsa S, Chan BP, Chua CL, Wijekoon N, de Silva HA, Hiyadan JH, Suwanwela NC, Wong KS, Poungvarin N, Eow GB, Chen CL; CHIMES-E Study Investigators. Prognostic Factors and Pattern of Long-Term Recovery with MLC601 (NeuroAiD™) in the Chinese Medicine NeuroAiD Efficacy on Stroke Recovery-Extension Study. Cerebrovasc Dis. 2017; 43 (1-2): 36-42. doi: 10.1159/000452285. Epub 2016 Nov 15. PMID: 27846631; PMCID: PMC5348731.	No data
Sommer P, Seyfang L, Posekany A, Ferrari J, Lang W, Fertl E, Serles W, Töll T, Kiechl S, Greisenegger S. Prehospital and intra-hospital time delays in posterior circulation stroke: results from the Austrian Stroke Unit Registry. J Neurol. 2017 Jan; 264 (1): 131-138. doi: 10.1007/s00415-016-8330-x. Epub 2016 Nov 7. PMID: 27822599; PMCID: PMC5225195.	Very specific population (posterior circulation strokes)
Correia M, Magalhães R, Felgueiras R, Quintas C, Guimarães L, Silva MC. Changes in stroke incidence, outcome, and associated factors in Porto between 1998 and 2011. Int J Stroke. 2017 Feb; 12 (2): 169-179. doi: 10.1177/1747493016669846. Epub 2016 Sep 30. PMID: 27694314.	No data
Lodi Y, Reddy V, Petro G, Devasenapathy A, Hourani A, Chou CA. Primary acute stroke thrombectomy within 3 h for large artery occlusion (PAST3-LAO): a pilot study. J Neurointerv Surg. 2017 Apr; 9 (4): 352-356. doi: 10.1136/neurintsurg-2015-012172. Epub 2016 Apr 11. PMID: 27067715.	No data
Myint PK, Kidd AC, Kwok CS, Musgrave SD, Redmayne O, Metcalf AK, Ngeh J, Nicolson A, Owusu-Agyei P, Shekhar R, Walsh K, Day DJ, Warburton EA, Bachmann MO, Potter JF; Anglia Stroke Clinical Network Evaluation Study (ASCNES) Group. Time to Computerized Tomography Scan, Age, and Mortality in Acute Stroke. J Stroke Cerebrovasc Dis. 2016 Dec; 25 (12): 3005-3012. doi: 10.1016/j.jstrokecerebrovasdis.2016.08.020. Epub 2016 Sep 8. PMID: 27618197.	No data
Pidaparthi L, Kotha A, Aleti VR, Kohat AK, Kandadai MR, Turaga S, Shaik JA, Alladi S, Kanikannan MA, Rupam B, Kaul S. Factors influencing nonadministration of thrombolytic therapy in early arrival strokes in a university hospital in Hyderabad, India. Ann Indian Acad Neurol. 2016 Jul-Sep; 19 (3): 351-5. doi: 10.4103/0972-2327.179976. PMID: 27570387; PMCID: PMC4980958.	No data
Sakamoto Y, Tanabe M, Masuda K, Ozaki H, Okubo S, Suda S, Abe A, Aoki J, Muraga K, Kanamaru T, Suzuki K, Katano T, Kimura K. Feasibility of using magnetic resonance imaging as a screening tool for acute stroke thrombolysis. J Neurol Sci. 2016 Sep 15; 368: 168-72. doi: 10.1016/j.jns.2016.07.011. Epub 2016 Jul 9. PMID: 27538625.	No data
Oostema JA, Carle T, Talia N, Reeves M. Dispatcher Stroke Recognition Using a Stroke Screening Tool: A Systematic Review. Cerebrovasc Dis. 2016; 42 (5-6): 370-377. doi: 10.1159/000447459. Epub 2016 Jun 28. PMID: 27348228.	No data
Mayasi Y, Helenius J, Goddeau RP Jr, Moonis M, Henninger N. Time to Presentation Is Associated with Clinical Outcome in Hemispheric Stroke Patients Deemed Ineligible for Recanalization Therapy. J Stroke Cerebrovasc Dis. 2016 Oct; 25 (10): 2373-9. doi: 10.1016/j.jstrokecerebrovasdis.2016.05.036. Epub 2016 Jun 14. PMID: 27315744; PMCID: PMC5026882.	No data
Le Bonniec A, Haesebaert J, Derex L, Porthault S, Préau M, Schott AM. Why Patients Delay Their First Contact with Health Services After Stroke? A Qualitative Focus Group-Based Study. PLoS One. 2016 Jun 8; 11 (6): e0156933. doi: 10.1371/journal.pone.0156933. PMID: 27275948; PMCID: PMC4898830.	No data
Menon BK, Sajobi TT, Zhang Y, Rempel JL, Shuaib A, Thornton J, Williams D, Roy D, Poppe AY, Jovin TG, Sapkota B, Baxter BW, Krings T, Silver FL, Frei DF, Fanale C, Tampieri D, Teitelbaum J, Lum C, Dowlatshahi D, Eesa M, Lowerison MW, Kamal NR, Demchuk AM, Hill MD, Goyal M. Analysis of Workflow and Time to Treatment on Thrombectomy Outcome in the Endovascular Treatment for Small Core and Proximal Occlusion Ischemic Stroke (ESCAPE) Randomized, Controlled Trial. Circulation. 2016 Jun 7; 133 (23): 2279-86. doi: 10.1161/CIRCULATIONAHA.115.019983. Epub 2016 Apr 13. PMID: 27076599.	No data
Wilson A, Coleby D, Regen E, Phelps K, Windridge K, Willars J, Robinson T. Service factors causing delay in specialist assessment for TIA and minor stroke: a qualitative study of GP and patient perspectives. BMJ Open. 2016 May 17; 6 (5): e011654. doi: 10.1136/bmjopen-2016-011654. PMID: 27188815; PMCID: PMC4874118.	No data
Gumbinger C, Reuter B, Hacke W, Sauer T, Bruder I, Diehm C, Wiethölter H, Schoser K, Daffertshofer M, Neumaier S, Drewitz E, Rode S, Kern R, Hennerici MG, Stock C, Ringleb P. Restriction of therapy mainly explains lower thrombolysis rates in reduced stroke service levels. Neurology. 2016 May 24; 86 (21): 1975-83. doi: 10.1212/WNL.0000000000002695. Epub 2016 Apr 22. PMID: 27164674.	No data
Kazandjian C, Kretz B, Lemogne B, Aboa Eboulé C, Béjot Y, Steinmetz E. Influence of the type of cerebral infarct and timing of intervention in the early outcomes after carotid endarterectomy for symptomatic stenosis. J Vasc Surg. 2016 May; 63 (5): 1256-61. doi: 10.1016/j.jvs.2015.10.097. PMID: 27109793.	No data
Gutiérrez JM, Emery RJ, Parker SA, Jackson K, Grotta JC. Radiation Monitoring Results from the First Year of Operation of a Unique Ambulance-based Computed Tomography Unit for the Improved Diagnosis and Treatment of Stroke Patients. Health Phys. 2016 May; 110 (5 Suppl 2): S73-80. doi: 10.1097/HP.0000000000000502. PMID: 27023154.	No data
Yoo J, Song D, Baek JH, Lee K, Jung Y, Cho HJ, Yang JH, Cho HJ, Choi HY, Kim YD, Nam HS, Heo JH. Comprehensive code stroke program to reduce reperfusion delay for in-hospital stroke patients. Int J Stroke. 2016 Aug; 11 (6): 656-62. doi: 10.1177/1747493016641724. Epub 2016 Mar 25. PMID: 27016511.	No data
Ribo M, Molina CA, Cobo E, Cerdà N, Tomasello A, Quesada H, De Miquel MA, Millan M, Castaño C, Urra X, Sanroman L, Dàvalos A, Jovin T; REVASCAT Trial Investigators. Association Between Time to Reperfusion and Outcome Is Primarily Driven by the Time From Imaging to Reperfusion. Stroke. 2016 Apr; 47 (4): 999-1004. doi: 10.1161/STROKEAHA.115.011721. Epub 2016 Mar 8. PMID: 26956258.	No data
Schwartz J, Dreyer RP, Murugiah K, Ranasinghe I. Contemporary Prehospital Emergency Medical Services Response Times for Suspected Stroke in the United States. Prehosp Emerg Care. 2016 Sep-Oct; 20 (5): 560-5. doi: 10.3109/10903127.2016.1139219. Epub 2016 Mar 8. PMID: 26953776.	No data
Leung LY, Caplan LR. Factors Associated with Delay in Presentation to the Hospital for Young Adults with Ischemic Stroke. Cerebrovasc Dis. 2016; 42 (1-2): 10-4. doi: 10.1159/000443242. Epub 2016 Mar 9. PMID: 26953591.	Very specific population (young people)
Palazón-Cabanes B, López-Picazo Ferrer JJ, Morales-Ortiz A, Tomás-García N. Identificación de los factores condicionantes de tiempos e indicadores de calidad en la atención intrahospitalaria al ictus agudo [Identification of the factors conditioning times and indicators of quality in the intrahospital care of acute stroke]. Rev Neurol. 2016 Feb 16; 62 (4): 157-64. Spanish. PMID: 26860720.	No data
Mellon L, Doyle F, Williams D, Brewer L, Hall P, Hickey A. Patient behaviour at the time of stroke onset: a cross-sectional survey of patient response to stroke symptoms. Emerg Med J. 2016 Jun; 33 (6): 396-402. doi: 10.1136/emermed-2015-204806. Epub 2016 Jan 18. PMID: 26781460.	No data
Zock E, Kerkhoff H, Kleyweg RP, van de Beek D. Intrinsic factors influencing help-seeking behaviour in an acute stroke situation. Acta Neurol Belg. 2016 Sep; 116 (3): 295-301. doi: 10.1007/s13760-015-0555-4. Epub 2016 Jan 5. PMID: 26732617; PMCID: PMC4989004.	No data
Brown AT, Wei F, Culp WC, Brown G, Tyler R, Balamurugan A, Bianchi N. Emergency transport of stroke suspects in a rural state: opportunities for improvement. Am J Emerg Med. 2016 Aug; 34 (8): 1640-4. doi: 10.1016/j.ajem.2016.06.044. Epub 2016 Jun 14. PMID: 27344100; PMCID: PMC4998736.	No data
Grunwald IQ, Ragoschke-Schumm A, Kettner M, Schwindling L, Roumia S, Helwig S, Manitz M, Walter S, Yilmaz U, Greveson E, Lesmeister M, Reith W, Fassbender K. First Automated Stroke Imaging Evaluation via Electronic Alberta Stroke Program Early CT Score in a Mobile Stroke Unit. Cerebrovasc Dis. 2016; 42 (5-6): 332-338. doi: 10.1159/000446861. Epub 2016 Jun 16. PMID: 27304197.	No data
Goyal M, Jadhav AP, Bonafe A, Diener H, Mendes Pereira V, Levy E, Baxter B, Jovin T, Jahan R, Menon BK, Saver JL; SWIFT PRIME investigators. Analysis of Workflow and Time to Treatment and the Effects on Outcome in Endovascular Treatment of Acute Ischemic Stroke: Results from the SWIFT PRIME Randomized Controlled Trial. Radiology. 2016 Jun; 279 (3): 888-97. doi: 10.1148/radiol.2016160204. Epub 2016 Apr 19. PMID: 27092472.	No data
Denti L, Artoni A, Scoditti U, Gatti E, Bussolati C, Ceda GP. Pre-hospital Delay as Determinant of Ischemic Stroke Outcome in an Italian Cohort of Patients Not Receiving Thrombolysis. J Stroke Cerebrovasc Dis. 2016 Jun; 25 (6): 1458-66. doi: 10.1016/j.jstrokecerebrovasdis.2016.02.032. Epub 2016 Mar 24. PMID: 27019987.	No data
Golden AP, Odoi A. Emergency medical services transport delays for suspected stroke and myocardial infarction patients. BMC Emerg Med. 2015 Dec 3; 15: 34. doi: 10.1186/s12873-015-0060-3. PMID: 26634914; PMCID: PMC4668620.	No data
Fransen PS, Berkhemer OA, Lingsma HF, Beumer D, van den Berg LA, Yoo AJ, Schonewille WJ, Vos JA, Nederkoorn PJ, Wermer MJ, van Walderveen MA, Staals J, Hofmeijer J, van Oostayen JA, Lycklama À Nijeholt GJ, Boiten J, Brouwer PA, Emmer BJ, de Bruijn SF, van Dijk LC, Kappelle LJ, Lo RH, van Dijk EJ, de Vries J, de Kort PL, van den Berg JS, van Hasselt BA, Aerden LA, Dallinga RJ, Visser MC, Bot JC, Vroomen PC, Eshghi O, Schreuder TH, Heijboer RJ, Keizer K, Tielbeek AV, den Hertog HM, Gerrits DG, van den Berg-Vos RM, Karas GB, Steyerberg EW, Flach HZ, Marquering HA, Sprengers ME, Jenniskens SF, Beenen LF, van den Berg R, Koudstaal PJ, van Zwam WH, Roos YB, van Oostenbrugge RJ, Majoie CB, van der Lugt A, Dippel DW; Multicenter Randomized Clinical Trial of Endovascular Treatment of Acute Ischemic Stroke in the Netherlands Investigators. Time to Reperfusion and Treatment Effect for Acute Ischemic Stroke: A Randomized Clinical Trial. JAMA Neurol. 2016 Feb; 73 (2): 190-6. doi: 10.1001/jamaneurol.2015.3886. Erratum in: JAMA Neurol. 2016 Apr; 73 (4): 481. PMID: 26716735.	No data
Skolarus LE, Zimmerman MA, Bailey S, Dome M, Murphy JB, Kobrossi C, Dombrowski SU, Burke JF, Morgenstern LB. Stroke Ready Intervention: Community Engagement to Decrease Prehospital Delay. J Am Heart Assoc. 2016 May 20; 5 (5): e003331. doi: 10.1161/JAHA.116.003331. PMID: 27208000; PMCID: PMC4889198.	No data
Meurer WJ, Levine DA, Kerber KA, Zahuranec DB, Burke J, Baek J, Sánchez B, Smith MA, Morgenstern LB, Lisabeth LD. Neighborhood Influences on Emergency Medical Services Use for Acute Stroke: A Population-Based Cross-sectional Study. Ann Emerg Med. 2016 Mar; 67 (3): 341-348.e4. doi: 10.1016/j.annemergmed.2015.07.524. Epub 2015 Sep 16. PMID: 26386884; PMCID: PMC4769663.	No data
McKay C, Hall AB, Cortes J. Time to Blood Pressure Control Before Thrombolytic Therapy in Patients With Acute Ischemic Stroke: Comparison of Labetalol, Nicardipine, and Hydralazine. J Neurosci Nurs. 2015 Dec; 47 (6): 327-32. doi: 10.1097/JNN.0000000000000170. PMID: 26528950.	No data
Wang G, Liu G, Zhang R, Ji R, Gao B, Wang Y, Pan Y, Li Z, Wang Y. Evaluation of Off-Hour Emergency Care in Acute Ischemic Stroke: Results from the China National Stroke Registry. PLoS One. 2015 Sep 17; 10 (9): e0138046. doi: 10.1371/journal.pone.0138046. PMID: 26379280; PMCID: PMC4574931.	No data
Lansberg MG, Cereda CW, Mlynash M, Mishra NK, Inoue M, Kemp S, Christensen S, Straka M, Zaharchuk G, Marks MP, Bammer R, Albers GW; Diffusion and Perfusion Imaging Evaluation for Understanding Stroke Evolution 2 (DEFUSE 2) Study Investigators. Response to endovascular reperfusion is not time-dependent in patients with salvageable tissue. Neurology. 2015 Aug 25; 85 (8): 708-14. doi: 10.1212/WNL.0000000000001853. Epub 2015 Jul 29. PMID: 26224727; PMCID: PMC4553034.	No data
Lin CB, Cox M, Olson DM, Britz GW, Constable M, Fonarow GC, Schwamm L, Peterson ED, Shah BR. Perception Versus Actual Performance in Timely Tissue Plasminogen Activation Administration in the Management of Acute Ischemic Stroke. J Am Heart Assoc. 2015 Jul 22; 4 (7): e001298. doi: 10.1161/JAHA.114.001298. PMID: 26201547; PMCID: PMC4608060.	No data
Sheth SA, Jahan R, Gralla J, Pereira VM, Nogueira RG, Levy EI, Zaidat OO, Saver JL; SWIFT-STAR Trialists. Time to endovascular reperfusion and degree of disability in acute stroke. Ann Neurol. 2015 Oct; 78 (4): 584-93. doi: 10.1002/ana.24474. Epub 2015 Aug 17. PMID: 26153450; PMCID: PMC4955570.	No data
Boden-Albala B, Stillman J, Roberts ET, Quarles LW, Glymour MM, Chong J, Moats H, Torrico V, Parides MC. Comparison of Acute Stroke Preparedness Strategies to Decrease Emergency Department Arrival Time in a Multiethnic Cohort: The Stroke Warning Information and Faster Treatment Study. Stroke. 2015 Jul; 46 (7): 1806-12. doi: 10.1161/STROKEAHA.114.008502. Epub 2015 Jun 11. PMID: 26069259.	No data
Yoo J, Song D, Lee K, Kim YD, Nam HS, Heo JH. Comparison of Outcomes after Reperfusion Therapy between In-Hospital and Out-of-Hospital Stroke Patients. Cerebrovasc Dis. 2015; 40 (1-2): 28-34. doi: 10.1159/000381787. Epub 2015 Jun 10. PMID: 26068226.	No data
Philip-Ephraim EE, Charidimou A, Otu AA, Eyong EK, Williams UE, Ephraim RP. Factors associated with prehospital delay among stroke patients in a developing African country. Int J Stroke. 2015 Jun; 10 (4): E39. doi: 10.1111/ijs.12469. PMID: 25973710.	No data
Saltman AP, Silver FL, Fang J, Stamplecoski M, Kapral MK. Care and Outcomes of Patients With In-Hospital Stroke. JAMA Neurol. 2015 Jul; 72 (7): 749-55. doi: 10.1001/jamaneurol.2015.0284. PMID: 25938195.	Very specific population (in-hospital stroke)
Sauser K, Bravata DM, Hayward RA, Levine DA. A National Evaluation of Door-to-Imaging Times among Acute Ischemic Stroke Patients within the Veterans Health Administration. J Stroke Cerebrovasc Dis. 2015 Jun; 24 (6): 1329-32. doi: 10.1016/j.jstrokecerebrovasdis.2015.02.007. Epub 2015 Apr 13. PMID: 25881775.	No data
Klein KE, Rasmussen PA, Winners SL, Frontera JA. Teleneurocritical care and telestroke. Crit Care Clin. 2015 Apr; 31 (2): 197-224. doi: 10.1016/j.ccc.2014.12.002. PMID: 25814450.	No data
Yanagida T, Fujimoto S, Inoue T, Suzuki S. Prehospital delay and stroke-related symptoms. Intern Med. 2015; 54 (2): 171-7. doi: 10.2169/internalmedicine.54.2684. Epub 2015 Jan 15. PMID: 25743008.	No data
Mellor RM, Bailey S, Sheppard J, Carr P, Quinn T, Boyal A, Sandler D, Sims DG, Mant J, Greenfield S, McManus RJ. Decisions and delays within stroke patients’ route to the hospital: a qualitative study. Ann Emerg Med. 2015 Mar; 65 (3): 279-287.e3. doi: 10.1016/j.annemergmed.2014.10.018. Epub 2014 Nov 15. PMID: 25455907.	No data
Asuzu D, Nyström K, Amin H, Schindler J, Wira C, Greer D, Chi NF, Halliday J, Sheth KN. On- versus Off-Hour Patient Cohorts at a Primary Stroke Center: Onset-to-Treatment Duration and Clinical Outcomes after IV Thrombolysis. J Stroke Cerebrovasc Dis. 2016 Feb; 25 (2): 447-51. doi: 10.1016/j.jstrokecerebrovasdis.2015.10.017. Epub 2015 Nov 30. PMID: 26654664	No data
Laurencin C, Philippeau F, Blanc-Lasserre K, Vallet AE, Cakmak S, Mechtouff L, Cho TH, Ritzenthaler T, Flocard E, Bischoff M, El Khoury C, Nighoghossian N, Derex L. Thrombolysis for Acute Minor Stroke: Outcome and Barriers to Management. Results from the RESUVAL Stroke Network. Cerebrovasc Dis. 2015; 40 (1-2): 3-9. doi: 10.1159/000381866. Epub 2015 May 14. PMID: 25998791.	Very specific population (minor stroke)
He AH, Churilov L, Mitchell PJ, Dowling RJ, Yan B. Every 15-min delay in recanalization by intra-arterial therapy in acute ischemic stroke increases risk of poor outcome. Int J Stroke. 2015 Oct; 10 (7): 1062-7. doi: 10.1111/ijs.12495. Epub 2015 Apr 28. PMID: 25918863.	No data
Dorňák T, Herzig R, Kuliha M, Havlíček R, Školoudík D, Šaňák D, Köcher M, Procházka V, Lacman J, Charvát F, Krajina A, Krajíčková D, Král M, Veverka T, Roubec M, Hajduková L, Zapletalová J. Endovascular treatment of acute basilar artery occlusion: time to treatment is crucial. Clin Radiol. 2015 May; 70 (5): e20-7. doi: 10.1016/j.crad.2015.01.008. Epub 2015 Feb 19. PMID: 25703459.	No data
Rodríguez-Rivera IV, Santiago F, Estapé ES, González-Sepúlveda L, Brau R. Impact of Day of the Week and Time of Arrival on Ischemic Stroke Management. P R Health Sci J. 2015 Sep; 34 (3): 164-9. PMID: 26356742; PMCID: PMC4633410.	No data
Alexandra P. Saltman, MD; Frank L. Silver, MD; Jiming Fang, PhD; Melissa Stamplecoski, BSc; Moira K. Kapral, MD, MSc. Care and Outcomes of Patients With In-Hospital Stroke.jamaneurol.2015.0284	No data
Shreyansh Shah,; Ying Xian,; Shubin Sheng, Kori S. Zachrison; Jeffrey L. Saver; Kevin N. Sheth, Gregg C. Fonarow, Lee H. Schwamm, Eric E. Smith. Use, Temporal Trends, and Outcomes of Endovascular Therapy after Interhospital Transfer in the United States. 2012.10.1161/CIRCULATIONAHA.118.036509	No data

**Table A2:** Quality assessment of studies included in the systematic literature review based on *The Newcastle-Ottawa Scale instrument*.

Cross-Sectional Studies (Green)	Selection (≤3)				Comparability (≤2)		Results (≤2)				Total
	A1	A2	A3	A4	B1	B2	C1	C2	C3	-	
Case Control Studies (Grey)	Selection (≤4)				Comparability (≤2)		Exposition (≤4)				
	A1	A2	A3	A4	B1a	B1b	C1	C2	C3	C4	
Ungerer MN, Busetto L, Begli NH, Riehle K, Regula J, Gumbinger C. Factors affecting prehospital delay in rural and urban patients with stroke: a prospective survey-based study in Southwest Germany. BMC Neurol. 2020.	*	*	*	*	*	*		*	*		8
Le SM, Copeland LA, Zeber JE, Benge JF, Allen L, Cho J, Liao IC, Rasmussen J. Factors affecting time between symptom onset and emergency department arrival in stroke patients. eNeurologicalSci. 2020.	*	*	*	*	*	*	*	*	*		9
Nagao Y, Nakajima M, Inatomi Y, Ito Y, Kouzaki Y, Wada K, Yonehara T, Terasaki T, Hashimoto Y, Ando Y. Pre-Hospital Delay in Patients with Acute Ischemic Stroke in a Multicenter Stroke Registry: K-PLUS. J Stroke Cerebrovasc Dis. 2020.	*	*	*	*	*	*		*	*		8
Rynor H, Levine J, Souchak J, Shashoua N, Ramirez M, Gonzalez IC, Ramos V, Saxena A, Veledar E, Starosciak AK, Rios La Rosa FL. The Effect of a County Prehospital FAST-ED Initiative on Endovascular Treatment Times. J Stroke Cerebrovasc Dis. 2020.	*	*	*	*	*	*		*	*		8
Noone ML, Moideen F, Krishna RB, Pradeep Kumar VG, Karadan U, Chellenton J, Salam KA. Mobile App Based Strategy Improves Door-to-Needle Time in the Treatment of Acute Ischemic Stroke. J Stroke Cerebrovasc Dis. 2020.	*	*	*	*	*	*		*	*	*	9
Kielkopf M, Meinel T, Kaesmacher J, Fischer U, Arnold M, Heldner M, Seiffge D, Mordasini P, Dobrocky T, Piechowiak E, Gralla J, Jung S. Temporal Trends and Risk Factors for Delayed Hospital Admission in Suspected Stroke Patients. J Clin Med. 2020.	*	*		*	*	*		*	*		7
Xu H, Xian Y, Woon FP, Bettger JP, Laskowitz DT, Ng YY, Ong MEH, Matchar DB, De Silva DA. Emergency medical services use and its association with acute ischaemic stroke evaluation and treatment in Singapore. Stroke Vasc Neurol. 2020.	*	*		*	*	*		*	*		7
Darehed D, Blom M, Glader EL, Niklasson J, Norrving B, Eriksson M. In-Hospital Delays in Stroke Thrombolysis: Every Minute Counts. Stroke. 2020.	*	*	*	*	*	*	*	*	*		9
Miyamoto Y, Aso S, Iwagami M, Morita K, Fushimi K, Hamasaki Y, Nangaku M, Doi K, Yasunaga H. Expanded Indication for Recombinant Tissue Plasminogen Activator from 3 to 4.5 h after Onset of Stroke in Japan. J Stroke Cerebrovasc Dis. 2020.	*	*		*	*	*		*	*		7
Al Khathaami AM, Al Bdah B, Tarawneh M, Alskaini M, Alotaibi F, Alshalan A, Almuhraj M, Aldaham D, Alotaibi N In. Utilization of intravenous Tissue Plasminogen Activator and Reasons for Non use in Acute Ischemic Stroke in Saudi Arabia. J Stroke Cerebrovasc Dis. 2020.		*		*	*	*			*		5
Zhu Y, Zhang X, You S, Cao X, Wang X, Gong W, Qin Y, Huang X, Cao Y, Shi R. Factors Associated with Pre-Hospital Delay and Intravenous Thrombolysis in China. J Stroke Cerebrovasc Dis. 2020.	*	*		*	*	*		*	*		7
Mashni SK, O’Neal CR, Abner E, Lee J, Fraser JF. Time Intervals for Direct Versus Transfer Cases of Thrombectomy for Stroke in a Primarily Rural System of Care. J Stroke Cerebrovasc Dis. 2020.	*	*	*		*	*		*		*	8
Sloane B, Bosson N, Sanossian N, Saver JL, Perez L, Gausche-Hill M. Is Door-to-Needle Time Reduced for Emergency Medical Services Transported Stroke Patients Routed Directly to the Computed Tomography Scanner on Emergency Department Arrival? J Stroke Cerebrovasc Dis. 2020.	*		*	*	*	*		*	*		8
Sharma R, Zachrison KS, Viswanathan A, Matiello M, Estrada J, Anderson CD, Etherton M, Silverman S, Rost NS, Feske SK, Schwamm LH. Trends in Telestroke Care Delivery: A 15-Year Experience of an Academic Hub and Its Network of Spokes. Circ Cardiovasc Qual Outcomes. 2020.	*	*		*	*	*	*	*	*		8
Kummer BR, Lerario MP, Hunter MD, Wu X, Efraim ES, Salehi Omran S, Chen ML, Diaz IL, Sacchetti D, Lekic T, Kulick ER, Pishanidar S, Mir SA, Zhang Y, Asaeda G, Navi BB, Marshall RS, Fink ME. Geographic Analysis of Mobile Stroke Unit Treatment in a Dense Urban Area: The New York City METRONOME Registry. J Am Heart Assoc. 2019.	*	*		*	*	*		*	*		7
Sanjuan Menéndez E, Girón Espot P, Calleja Macho L, Rodríguez-Samaniego MT, Santana Román KE, Rubiera Del Fueyo M. Implementation of a protocol for direct stroke patient transfer and mobilization of a stroke team to reduce times to reperfusion. Emergencias. 2019.	*		*	*		*		*	*	*	7
Soto-Cámara R, González-Santos J, González-Bernal J, Martín-Santidrian A, Cubo E, Trejo-Gabriel-Galán JM. Factors Associated with Shortening of Prehospital Delay among Patients with Acute Ischemic Stroke. J Clin Med. 2019.	*	*		*		*		*	*		6
Madhok DY, Keenan KJ, Cole SB, Martin C, Hemphill JC 3rd. Prehospital and Emergency Department-Focused Mission Protocol Improves Thrombolysis Metrics for Suspected Acute Stroke Patients. J Stroke Cerebrovasc Dis. 2019.	*	*		*	*	*	*	*		*	8
Fladt J, Meier N, Thilemann S, Polymeris A, Traenka C, Seiffge DJ, Sutter R, Peters N, Gensicke H, Flückiger B, de Hoogh K, Künzli N, Bringolf-Isler B, Bonati LH, Engelter ST, Lyrer PA, De Marchis GM. Reasons for Prehospital Delay in Acute Ischemic Stroke. J Am Heart Assoc. 2019.	*	*	*	*	*	*		*	*		7
Bonadio W, Beck C, Mueller A. Impact of CT scanner location on door to imaging time for emergency department stroke evaluation. Am J Emerg Med. 2020.	*	*		*	*	*	*		*	*	8
Choi PMC, Tsoi AH, Pope AL, Leung S, Frost T, Loh PS, Chandra RV, Ma H, Parsons M, Mitchell P, Dewey HM. Door-in-Door-Out Time of 60 Minutes for Stroke With Emergent Large Vessel Occlusion at a Primary Stroke Center. Stroke. 2019.	*		*	*		*		*	*	*	7
Jagolino-Cole AL, Bozorgui S, Ankrom CM, Vahidy F, Bambhroliya AB, Randhawa J, Trevino AD, Cossey TC, Savitz SI, Wu TC. Variability and Delay in Telestroke Physician Alert among Spokes in a Telestroke Network: A Need for Metric Benchmarks. J Stroke Cerebrovasc Dis. 2019.	*	*		*	*	*	*	*	*		8
Dimitriou P, Tziomalos K, Christou K, Kostaki S, Angelopoulou SM, Papagianni M, Ztriva E, Chatzopoulos G, Savopoulos C, Hatzitolios AI. Factors associated with delayed presentation at the emergency department in patients with acute ischemic stroke. Brain Inj. 2019.	*	*		*	*	*		*	*		7
Nepal G, Yadav JK, Basnet B, Shrestha TM, Kharel G, Ojha R. Status of prehospital delay and intravenous thrombolysis in the management of acute ischemic stroke in Nepal. BMC Neurol. 2019.	*	*		*	*	*		*	*		7
Gonzalez-Aquines A, Cordero-Pérez AC, Cristobal-Niño M, Pérez-Vázquez G, Góngora-Rivera F; GECEN Investigators. Contribution of Onset-to-Alarm Time to Prehospital Delay in Patients with Ischemic Stroke. J Stroke Cerebrovasc Dis. 2019.		*		*	*			*	*		5
Bhaskar S, Thomas P, Cheng Q, Clement N, McDougall A, Hodgkinson S, Cordato D. Trends in acute stroke presentations to an emergency department: implications for specific communities in accessing acute stroke care services. Postgrad Med J. 2019.	*	*		*	*	*	*	*	*		8
Chai E, Li C, Jiang L. Factors affecting in-hospital delay of intravenous thrombolysis for acute ischemic stroke: A retrospective cohort study. Medicine (Baltimore). 2019.		*		*	*			*	*		5
Weisenburger-Lile D, Blanc R, Kyheng M, Desilles JP, Labreuche J, Piotin M, Mazighi M, Consoli A, Lapergue B, Gory B; on behalf of the Endovascular Treatment in Ischemic Stroke Investigators. Direct Admission versus Secondary Transfer for Acute Stroke Patients Treated with Intravenous Thrombolysis and Thrombectomy: Insights from the Endovascular Treatment in Ischemic Stroke Registry. Cerebrovasc Dis. 2019.	*		*	*		*		*	*	*	7
Varjoranta T, Raatiniemi L, Majamaa K, Martikainen M, Liisanantti JH. Prehospital and hospital delays for stroke patients treated with thrombolysis: A retrospective study from mixed rural-urban area in Northern Finland. Australas Emerg Care. 2019.	*	*		*	*	*		*	*		7
Han TS, Gulli G, Affley B, Fluck D, Fry CH, Barrett C, Kakar P, Sharma S, Sharma P. New evidence-based A1, A2, A3 alarm time zones for transferring thrombolysed patients to hyper-acute stroke units: faster is better. Neurol Sci. 2019.	*	*	*	*		*	*	*	*		8
Manners J, Khandker N, Barron A, Aziz Y, Desai SM, Morrow B, Delfyett WT, Martin-Gill C, Shutter L, Jovin TG, Jadhav AP. An interdisciplinary approach to inhospital stroke improves stroke detection and treatment time. J Neurointerv Surg. 2019 Nov; 11 (11): 1080-1084. doi: 10.1136/neurintsurg-2019-014890. Epub 2019.	*			*		*		*	*	*	6
Nordanstig A, Palaszewski B, Asplund K, Norrving B, Wahlgren N, Wester P, Jood K, Rosengren L. Evaluation of the Swedish National Stroke Campaign: A population-based time-series study. Int J Stroke. 2019.	*	*	*	*	*	*		*	*	*	9
Gu HQ, Rao ZZ, Yang X, Wang CJ, Zhao XQ, Wang YL, Liu LP, Wang CY, Liu C, Li H, Li ZX, Xiao RP, Wang YJ; Chinese Stroke Center Alliance Investigators. Use of Emergency Medical Services and Timely Treatment Among Ischemic Stroke. Stroke. 2019	*	*	*	*	*	*	*	*	*		9
Faiz KW, Sundseth A, Thommessen B, Rønning OM. The knowing-doing gap in acute stroke-Does stroke knowledge translate into action? Brain Behav. 2019.		*	*	*	*			*	*	*	7
Jung S, Rosini JM, Nomura JT, Caplan RJ, Raser-Schramm J. Even Faster Door-to-Alteplase Times and Associated Outcomes in Acute Ischemic Stroke. J Stroke Cerebrovasc Dis. 2019.	*	*	*		*		*	*		*	7
Sobral S, Taveira I, Seixas R, Vicente AC, Duarte J, Goes AT, Durán D, Lopes J, Rita H, Nzwalo H. Late Hospital Arrival for Thrombolysis after Stroke in Southern Portugal: Who Is at Risk? J Stroke Cerebrovasc Dis. 2019.	*	*	*	*	*		*	*		*	8
Klingner C, Günther A, Brodoehl S, Witte OW, Klingner CM. Talk About Thrombolysis. Regular Case-Based Discussions of Stroke Thrombolysis Improve Door-to-Needle Time by 20. J Stroke Cerebrovasc Dis. 2019.		*	*	*	*			*	*	*	7
Kamal N, Shand E, Swanson R, Hill MD, Jeerakathil T, Imoukhuede O, Heinrichs I, Bakker J, Stoyberg C, Fowler L, Duckett S, Holsworth S, Mann B, Valaire S, Bestard J. Reducing Door-to-Needle Times for Ischaemic Stroke to a Median of 30 Minutes at a Community Hospital. Can J Neurol Sci. 2019.	*	*	*	*		*	*		*	*	8
Mourand I, Malissart P, Dargazanli C, Nogue E, Bouly S, Gaillard N, Boukriche Y, Corti L, Picot MC, Beaufils O, Chbicheb M, Sablot D, Bonafe A, Costalat V, Arquizan C. A Regional Network Organization for Thrombectomy for Acute Ischemic Stroke in the Anterior Circulation; Timing, Safety, and Effectiveness. J Stroke Cerebrovasc Dis. 2019.	*		*	*		*		*	*	*	7
Menon BK, Xu H, Cox M, Saver JL, Goyal M, Peterson E, Xian Y, Matsuoka R, Jehan R, Yavagal D, Gupta R, Mehta B, Bhatt DL, Fonarow GC, Schwamm LH, Smith EE. Components and Trends in Door to Treatment Times for Endovascular Therapy in Get With The Guidelines-Stroke Hospitals. Circulation. 2019.	*	*		*	*	*	*	*	*		8
Heikkilä I, Kuusisto H, Holmberg M, Palomäki A. Fast Protocol for Treating Acute Ischemic Stroke by Emergency Physicians. Ann Emerg Med. 2019		*	*		*	*	*	*	*		8
Hassankhani H, Soheili A, Vahdati SS, Mozaffari FA, Fraser JF, Gilani N. Treatment Delays for Patients With Acute Ischemic Stroke in an Iranian Emergency Department: A Retrospective Chart Review. Ann Emerg Med. 2019.	*	*		*	*			*	*		6
Olascoaga Arrate A, Freijo Guerrero MM, Fernández Maiztegi C, Azkune Calle I, Silvariño Fernández R, Fernández Rodríguez M, Vazquez Naveira P, Anievas Elena A, Iturraspe González I, Pérez Díez Y, Ruiz Fernández R. Use of emergency medical transport and impact on time to care in patients with ischaemic stroke. Neurologia. 2019.		*	*	*	*			*	*		6
Tong X, Wiltz JL, George MG, Odom EC, Coleman King SM, Chang T, Yin X; Paul Coverdell National Acute Stroke Program team, Merritt RK. A Decade of Improvement in Door-to-Needle Time Among Acute Ischemic Stroke Patients, 2008 to 2017. Circ Cardiovasc Qual Outcomes. 2018.		*		*	*	*	*	*	*		7
Hebant B, Triquenot-Bagan A, Guegan-Massardier E, Ozkul-Wermester O, Maltête D. In-hospital delays to stroke thrombolysis: Out of hours versus regular hours and reduction in treatment times through the creation of a 24/7 mobile thrombolysis team. J Neurol Sci. 2018.	*	*		*	*	*	*	*	*		8
Cone DC, Cooley C, Ferguson J, Harrell AJ, Luk JH, Martin-Gill C, Marquis SW, Pasichow S. Observational Multicenter Study of a Direct-to-CT Protocol for EMS-transported Patients with Suspected Stroke. Prehosp Emerg Care. 2018.	*			*	*	*	*	*		*	7
Al Khathaami AM, Mohammad YO, Alibrahim FS, Jradi HA. Factors associated with late arrival of acute stroke patients to emergency department in Saudi Arabia. SAGE Open Med. 2018.		*	*	*	*			*	*		6
Moreno A., Schwamm LH, Siddiqui KA, Viswanathan A, Whitney C, Rost N, Zachrison KS. Frequent Hub-Spoke Contact Is Associated with Improved Spoke Hospital Performance: Results from the Massachusetts General Hospital Telestroke Network. Telemed J E Health. 2018.	*		*	*	*	*			*	*	7
Gonzalez-Aquines A, Cordero-Perez AC, Ramirez-Martinez LA, Sanchez-Teran H, Escobedo-Zuñiga N, Treviño-Herrera AB, Gongora-Rivera F, Pérez-Vázquez G, Contreras-Salazar K, Gómez-Padilla D. Onset-to-alarm time in patients with acute stroke: Results from a Mexican population. Int J Stroke. 2018.	*	*	*	*	*	*		*	*	*	9
Man S, Zhao X, Uchino K, Hussain MS, Smith EE, Bhatt DL, Xian Y, Schwamm LH, Shah S, Khan Y, Fonarow GC. Comparison of Acute Ischemic Stroke Care and Outcomes Between Comprehensive Stroke Centers and Primary Stroke Centers in the United States. Circ Cardiovasc Qual Outcomes. 2018.	*	*		*	*	*	*	*	*		8
Bohmann FO, Tahtali D, Kurka N, Wagner M, You SJ, du Mesnil de Rochemont R, Berkefeld J, Hartmetz AK, Kuhlmann A, Lorenz MW, Schütz A, Kress B, Henke C, Tritt S, Meyding-Lamadé U, Steinmetz H, Pfeilschifter W. A Network-Wide Stroke Team Program Reduces Time to Treatment for Endovascular Stroke Therapy in a Regional Stroke-Network. Cerebrovasc Dis. 2018.	*	*		*	*	*	*	*	*		8
Springer MV, Labovitz DL. The Effect of Being Found with Stroke Symptoms on Predictors of Hospital Arrival. J Stroke Cerebrovasc Dis. 2018 May; 27 (5): 1363-1367. doi: 10.1016/j.jstrokecerebrovasdis.2017.12.024. Epub 2018 Feb 8. PMID: 29428327.		*	*		*			*	*		5
Andersson Hagiwara M, Wireklint Sundström B, Brink P, Herlitz J, Hansson PO. A shorter system delay for haemorrhagic stroke than ischaemic stroke among patients who use emergency medical service. Acta Neurol Scand. 2018 May; 137 (5): 523-530. doi: 10.1111/ane.12895. Epub 2018 Jan 8. PMID: 29315463.	*	*		*	*	*	*	*	*		8
Tan BYQ, Ngiam NJH, Sunny S, Kong WY, Tam H, Sim TB, Leong BSH, Bhartendu C, Paliwal PR, Seet RCS, Chan BPL, Teoh HL, Sharma VK, Yeo LLL. Improvement in Door-to-Needle Time in Patients with Acute Ischemic Stroke via a Simple Stroke Activation Protocol. J Stroke Cerebrovasc Dis. 2018.	*	*		*		*	*	*	*		7
Haesebaert J, Nighoghossian N, Mercier C, Termoz A, Porthault S, Derex L, Gueugniaud PY, Bravant E, Rabilloud M, Schott AM; AVC II Trial group. Improving Access to Thrombolysis and Inhospital Management Times in Ischemic Stroke: A Stepped-Wedge Randomized Trial. Stroke. 2018.	*	*	*	*		*	*		*	*	8
Nguyen-Huynh MN, Klingman JG, Avins AL, Rao VA, Eaton A, Bhopale S, Kim AC, Morehouse JW, Flint AC; KPNC Stroke FORCE Team. Novel Telestroke Program Improves Thrombolysis for Acute Stroke Across 21 Hospitals of an Integrated Healthcare System. Stroke. 2018.	*	*	*	*		*	*		*	*	8
Hsieh MJ, Chien KL, Sun JT, Tang SC, Tsai LK, Chiang WC, Chien YC, Jeng JS, Huei-Ming Ma M; Taipei EMS Stroke Collaborative Group. The effect and associated factors of dispatcher recognition of stroke: A retrospective observational study. J Formos Med Assoc. 2018.		*		*	*	*		*			6
García Ruiz R, Silva Fernández J, García Ruiz RM, Recio Bermejo M, Arias Arias Á, Del Saz Saucedo P, Huertas Arroyo R, González Manero A, Santos Pinto A, Navarro Muñoz S, Botia Paniagua E, Abellán Alemán J. Response to Symptoms and Prehospital Delay in Stroke Patients. Is It Time to Reconsider Stroke Awareness Campaigns? J Stroke Cerebrovasc Dis. 2018.		*		*	*	*	*	*	*		7
Al Kasab S, Harvey JB, Debenham E, Jones DJ, Turner N, Holmstedt CA. Door to Needle Time over Telestroke-A Comprehensive Stroke Center Experience. Telemed J E Health. 2018.	*	*	*	*	*	*			*	*	8
Lawrence E, Merbach D, Thorpe S, Llinas RH, Marsh EB. Streamlining the Process for Intravenous Tissue Plasminogen Activator. J Neurosci Nurs. 2018.	*	*	*	*	*					*	6
Denis Sablot, Ioana Ion, Khaled Khlifa, Geoffroy Farouil, Franck Leibinger, Nicolas Gaillard, Alexandre Laverdure, Zoubir Mourad Bensalah, Julie Mas, Bénédicte Fadat, Philippe Smadja, Adélaïde Ferraro-Allou, Jean-Marie Bonnec, Nadège Olivier, Anaïs Dutray~, Maxime Tardieu, Adrian Dumitrana, Aymeric Guibal, Snejana Jurici, Jean-Louis Bertrand, Thibaut Allou, Caroline Arquizan, Alain Bonafe. Target Door-to-Needle Time for Tissue Plasminogen Activator Treatment with Magnetic ResonanceImaging Screening Can Be Reduced to 45 min. Cerebrovasc Dis 2018.		*	*	*	*			*	*		6
Brown CW, Macleod MJ. The positive predictive value of an ambulance prealert for stroke and transient ischaemic attack. Eur J Emerg Med. 2018.	*	*	*	*	*			*	*		7
Ribo M, Boned S, Rubiera M, Tomasello A, Coscojuela P, Hernández D, Pagola J, Juega J, Rodriguez N, Muchada M, Rodriguez-Luna D, Molina CA. Direct transfer to angiosuite to reduce door-to-puncture time in thrombectomy for acute stroke. J Neurointerv Surg. 2018.	*	*		*	*	*		*	*	*	8
Abraham SV, Krishnan SV, Thaha F, Balakrishnan JM, Thomas T, Palatty BU. Factors delaying management of acute stroke: An Indian scenario. Int J Crit Illn Inj Sci. 2017.		*		*	*			*	*		5
Springer MV, Labovitz DL, Hochheiser EC. Race-Ethnic Disparities in Hospital Arrival Time after Ischemic Stroke. Ethn Dis. 2017.		*		*	*			*	*		5
Denti L, Caminiti C, Scoditti U, Zini A, Malferrari G, Zedde ML, Guidetti D, Baratti M, Vaghi L, Montanari E, Marcomini B, Riva S, Iezzi E, Castellini P, Olivato S, Barbi F, Perticaroli E, Monaco D, Iafelice I, Bigliardi G, Vandelli L, Guareschi A, Artoni A, Zanferrari C, Schulz PJ. Impact on Prehospital Delay of a Stroke Preparedness Campaign: A SW-RCT (Stepped-Wedge Cluster Randomized Controlled Trial). Stroke. 2017. ¥	*	*	*	*	*	*	*	*	*	*	10
Bray JE, Denisenko S, Campbell BCV, Stephenson M, Muller J, Hocking G, Hand PJ, Bladin CF. Strategic framework improves access to stroke reperfusion across the state of Victoria Australia. Intern Med J. 2017.	*	*	*		*	*		*	*	*	8
Iglesias Mohedano AM, García Pastor A, Díaz Otero F, Vázquez Alen P, Vales Montero M, Luque Buzo E, Redondo Ráfales N, Chavarria Cano B, Fernández Bullido Y, Villanueva Osorio JÁ, Gil Núñez A. Efficacy of New Measures Saving Time in Acute Stroke Management: A Quantified Analysis. J Stroke Cerebrovasc Dis. 2017.	*		*		*	*		*	*	*	7
Andrew BY, Stack CM, Yang JP, Dodds JA. mStroke: “Mobile Stroke”-Improving Acute Stroke Care with Smartphone Technology. J Stroke Cerebrovasc Dis. 2017.	*		*		*	*		*	*	*	7
Mowla A, Doyle J, Lail NS, Rajabzadeh-Oghaz H, Deline C, Shirani P, Ching M, Crumlish A, Steck DA, Janicke D, Levy EI, Sawyer RN. Delays in door-to-needle time for acute ischemic stroke in the emergency department: A comprehensive stroke center experience. J Neurol Sci. 2017.	*	*		*	*		*	*	*		7
Wireklint Sundström B, Andersson Hagiwara M, Brink P, Herlitz J, Hansson PO. The early chain of care and risk of death in acute stroke in relation to the priority given at the dispatch centre: A multicentre observational study. Eur J Cardiovasc Nurs.		*		*	*			*	*		5
Eriksson M, Glader EL, Norrving B, Stegmayr B, Asplund K. Acute stroke alert activation, emergency service use, and reperfusion therapy in Sweden. Brain Behav. 2017.	*	*		*	*		*	*	*		7
Hillen ME, He W, Al-Qudah Z, Wang W, Hidalgo A, Walia J. Long-Term Impact of Implementation of a Stroke Protocol on Door-to-Needle Time in the Administration of Intravenous Tissue Plasminogen Activator. J Stroke Cerebrovasc Dis. 2017.	*	*	*		*	*		*	*	*	8
Threlkeld ZD, Kozak B, McCoy D, Cole S, Martin C, Singh V. Collaborative Interventions Reduce Time-to-Thrombolysis for Acute Ischemic Stroke in a Public Safety Net Hospital. J Stroke Cerebrovasc Dis. 2017.	*	*	*	*	*	*	*			*	8
Liu Q, Ranta AA, Abernethy G, Barber PA. Trends in New Zealand stroke thrombolysis treatment rates. N Z Med J. 2017.	*	*		*	*			*	*	*	7
Taqui A, Cerejo R, Itrat A, Briggs FB, Reimer AP, Winners S, Organek N, Buletko AB, Sheikhi L, Cho SM, Buttrick M, Donohue MM, Khawaja Z, Wisco D, Frontera JA, Russman AN, Hustey FM, Kralovic DM, Rasmussen P, Uchino K, Hussain MS; Cleveland Pre-Hospital Acute Stroke Treatment (PHAST) Group. Reduction in time to treatment in prehospital telemedicine evaluation and thrombolysis. Neurology. 2017.	*	*	*	*	*	*	*	*	*	*	10
Khurana D, Das B, Kumar A, Kumar S A, Khandelwal N, Lal V, Prabhakar S. Temporal Trends in Intravenous Thrombolysis in Acute Ischemic Stroke: Experience from a Tertiary Care Center in India. J Stroke Cerebrovasc Dis. 2017.	*	*		*	*		*	*	*		7
Kamal N, Sheng S, Xian Y, Matsouaka R, Hill MD, Bhatt DL, Saver JL, Reeves MJ, Fonarow GC, Schwamm LH, Smith EE. Delays in Door-to-Needle Times and Their Impact on Treatment Time and Outcomes in Get With The Guidelines-Stroke. Stroke. 2017 Apr; 48 (4): 946-954. doi: 10.1161/STROKEAHA.116.015712. 2017.	*	*	*	*	*		*	*	*		8
Martin A. Reznek, MD, MBA; Evangelia Murray, MS; Marguerite N. Youngren, BA; Natassia T. Durham, MSIE; Sean S. Michael, MD. Door-to-Imaging Time for Acute Stroke Patients Is Adversely Affected by Emergency Department Crowding. Stroke. 2017; 48: 00-00.	*	*		*	*		*	*	*		7
Sablot D, Gaillard N, Colas C, Smadja P, Gely C, Dutray A, Bonnec JM, Jurici S, Farouil G, Ferraro-Allou A, Jantac M, Allou T, Pujol C, Olivier N, Laverdure A, Fadat B, Mas J, Dumitrana A, Garcia Y, Touzani H, Perucho P, Moulin T, Richard C, Heroum C, Bouly S, Sagnes-Raffy C, Heve D. Results of a 1-year quality-improvement process to reduce door-to-needle time in acute ischemic stroke with MRI screening. Rev Neurol (Paris). 2017.	*	*	*		*	*	*	*	*	*	9
Slivinski A, Jones R, Whitehead H, Hooper V. Improving Access to Stroke Care in the Rural Setting: The Journey to Acute Stroke Ready Designation. J Emerg Nurs. 2017.	*	*		*	*	*		*	*	*	7
Kamal N, Holodinsky JK, Stephenson C, Kashayp D, Demchuk AM, Hill MD, Vilneff RL, Bugbee E, Zerna C, Newcommon N, Lang E, Knox D, Smith EE. Improving Door-to-Needle Times for Acute Ischemic Stroke: Effect of Rapid Patient Registration, Moving Directly to Computed Tomography, and Giving Alteplase at the Computed Tomography Scanner. Circ Cardiovasc Qual Outcomes. 2017.		*	*	*	*	*	*	*	*		8
Xian Y, Xu H, Lytle B, Blevins J, Peterson ED, Hernandez AF, Smith EE, Saver JL, Messé SR, Paulsen M, Suter RE, Reeves MJ, Jauch EC, Schwamm LH, Fonarow GC. Use of Strategies to Improve Door-to-Needle Times With Tissue-Type Plasminogen Activator in Acute Ischemic Stroke in Clinical Practice: Findings from Target: Stroke.Circ Cardiovasc Qual Outcomes. 2017.	*		*	*	*	*	*	*		*	8
Puy L, Lamy C, Canaple S, Arnoux A, Laine N, Iacob E, Constans JM, Godefroy O. Creation of an intensive care unit and organizational changes in an adult emergency department: Impact on acute stroke management. Am J Emerg Med. 2017.	*	*		*	*	*	*		*	*	8
Chen BY, Moussaddy A, Keezer MR, Deschaintre Y, Poppe AY. Short- and Long-Term Reduction of Door-to-Needle Time in Thrombolysis for Acute Stroke. Can J Neurol Sci. 2017.	*		*	*		*	*	*		*	7
Reznek MA, Murray E, Youngren MN, Durham NT, Michael SS. Door-to-Imaging Time for Acute Stroke Patients Is Adversely Affected by Emergency Department Crowding. Stroke. 2017 Jan; 48 (1): 49-54. doi: 10.1161/STROKEAHA.116.015131. Epub 2016 Nov 17. PMID: 27856953.	*	*		*	*		*	*	*		7
Metts EL, Bailey AM, Weant KA, Justice SB. Identification of Rate-Limiting Steps in the Provision of Thrombolytics for Acute Ischemic Stroke. J Pharm Pract. 2017 Dec; 30 (6): 606-611. doi: 10.1177/0897190016674408. Epub 2016 Nov 10. PMID: 27834297.		*		*	*		*	*	*		6
Zhou Y, Yang T, Gong Y, Li W, Chen Y, Li J, Wang M, Yin X, Hu B, Lu Z. Pre-hospital Delay after Acute Ischemic Stroke in Central Urban China: Prevalence and Risk Factors. Mol Neurobiol. 2017.		*		*	*		*	*	*		6
Aghaebrahim A, Streib C, Rangaraju S, Kenmuir CL, Giurgiutiu DV, Horev A, Saeed Y, Callaway CW, Guyette FX, Martin-Gill C, Pacella C, Ducruet AF, Jankowitz BT, Jovin TG, Jadhav AP. Streamlining door to recanalization processes in endovascular stroke therapy. J Neurointerv Surg. 2017.	*	*		*	*	*	*		*		7
Siju V. Abraham, S. Vimal Krishnan, Fazil Thaha, Jayaraj Mymbilly Balakrishnan, Tom Thomas, and Babu Urumese Palatty. Factors delaying management of acute stroke: An Indian scenario. Int J Crit Illn Inj Sci. 2017.		*		*			*	*			4
Zinkstok SM, Beenen LF, Luitse JS, Majoie CB, Nederkoorn PJ, Roos YB. Thrombolysis in Stroke within 30 Minutes: Results of the Acute Brain Care Intervention Study. PLoS One. 2016.	*		*	*	*	*	*	*	*	*	9
Hubert GJ, Meretoja A, Audebert HJ, Tatlisumak T, Zeman F, Boy S, Haberl RL, Kaste M, Müller-Barna P. Stroke Thrombolysis in a Centralized and a Decentralized System (Helsinki and Telemedical Project for Integrative Stroke Care Network). Stroke. 2016 Dec; 47 (12): 2999-3004. doi: 10.1161/STROKEAHA.116.014258. Epub 2016.	*	*	*		*	*	*	*		*	8
Prabhakaran S, Lee J, O’Neill K. Regional Learning Collaboratives Produce Rapid and Sustainable Improvements in Stroke Thrombolysis Times. Circ Cardiovasc Qual Outcomes. 2016 Sep; 9 (5): 585-92. doi: 10.1161/CIRCOUTCOMES.116.003222. Epub 2016.		*		*	*	*	*		*	*	7
Belt GH, Felberg RA, Rubin J, Halperin JJ. In-Transit Telemedicine Speeds Ischemic Stroke Treatment: Preliminary Results. Stroke. 2016.	*	*		*	*		*	*	*	*	8
Park MS, Lee JS, Park TH, Cho YJ, Hong KS, Park JM, Kang K, Lee KB, Kim JG, Lee SJ, Lee J, Choi KH, Kim JT, Cho KH, Oh MS, Yu KH, Lee BC, Cha JK, Kim DH, Nah HW, Lee J, Kim DE, Ryu WS, Kim BJ, Han MK, Bae HJ, Song SK, Choi JC. Characteristics of the Drip-and-Ship Paradigm for Patients with Acute Ischemic Stroke in South Korea. J Stroke Cerebrovasc Dis. 2016.	*	*	*	*	*	*		*	*		8
Birnbaum LA, Rodriguez JS, Topel CH, Behrouz R, Misra V, Palacio S, Patterson MG, Motz DS, Goros MW, Cornell JE, Caron JR. Older Stroke Patients with High Stroke Scores Have Delayed Door-To-Needle Times. J Stroke Cerebrovasc Dis. 2016.	*	*		*	*		*	*	*		7
Marto JP, Borbinha C, Calado S, Viana-Baptista M. The Stroke Chronometer-A New Strategy to Reduce Door-to-Needle Time. J Stroke Cerebrovasc Dis. 2016.	*	*	*		*	*	*	*	*	*	9
Koch PM, Kunz A, Ebinger M, Geisler F, Rozanski M, Waldschmidt C, Weber JE, Wendt M, Winter B, Zieschang K, Bollweg K, Kaczmarek S, Endres M, Audebert HJ. Influence of Distance to Scene on Time to Thrombolysis in a Specialized Stroke Ambulance. Stroke. 2016.	*	*	*	*		*	*		*		7
Ibrahim F, Akhtar N, Salam A, Kamran S, Deleu D, D’Souza A, Imam Y, Bourke P, Joseph S, Santos M, Khan R, Bhutta ZA, Bhagat A, Shuaib A. Stroke Thrombolysis Protocol Shortens “Door-to-Needle Time” and Improves Outcomes-Experience at a Tertiary Care Center in Qatar. J Stroke Cerebrovasc Dis. 2016.	*		*		*		*	*	*	*	7
Huang Q, Song HQ, Ji XM, Cheng WY, Feng J, Wu J, Ma QF. Generalization of the Right Acute Stroke Prevention Strategies in Reducing in-Hospital Delays. PLoS One. 2016.	*	*		*	*		*	*	*		7
Puolakka T, Strbian D, Harve H, Kuisma M, Lindsberg PJ. Prehospital Phase of the Stroke Chain of Survival: A Prospective Observational Study. J Am Heart Assoc. 2016.	*	*	*	*	*	*		*	*		8
Vidale S, Arnaboldi M, Bezzi G, Bono G, Grampa G, Guidotti M, Perrone P, Salmaggi A, Zarcone D, Zoli A, Agostoni E; Northern Lombardy Emergency Stroke Study Group. Reducing time delays in the management of ischemic stroke patients in Northern Italy. Int J Cardiol. 2016.	*	*	*		*	*		*		*	7
Hsieh MJ, Tang SC, Chiang WC, Tsai LK, Jeng JS, Ma MH; Taipei EMS Stroke Collaborative Group. Effect of prehospital notification on acute stroke care: a multicenter study. Scand J Trauma Resusc Emerg Med. 2016.		*		*	*	*		*	*		6
Liang Z, Ren L, Wang T, Hu H, Li W, Wang Y, Liu D, Lie Y. Effective management of patients with acute ischemic stroke based on lean production on thrombolytic flow optimization. Australas Phys Eng Sci Med. 2016.	*	*			*			*		*	5
Kim DH, Nah HW, Park HS, Choi JH, Kang MJ, Huh JT, Cha JK. Impact of Prehospital Intervention on Delay Time to Thrombolytic Therapy in a Stroke Center with a Systemized Stroke Code Program. J Stroke Cerebrovasc Dis. 2016.	*	*	*		*	*		*		*	7
Advani R, Naess H, Kurz M. Mass Media Intervention in Western Norway Aimed at Improving Public Recognition of Stroke, Emergency Response, and Acute Treatment. J Stroke Cerebrovasc Dis. 2016.	*		*		*	*		*	*	*	7
Dickson RL, Sumathipala D, Reeves J. Stop Stroke© Acute Care Coordination Medical Application: A Brief Report on Postimplementation Performance at a Primary Stroke Center. J Stroke Cerebrovasc Dis. 2016.		*	*	*	*			*	*	*	7
Groot AE, van Schaik IN, Visser MC, Nederkoorn PJ, Limburg M, Aramideh M, de Beer F, Zwetsloot CP, Halkes P, de Kruijk J, Kruyt ND, van der Meulen W, Spaander F, van der Ree T, Kwa VI, Van den Berg-Vos RM, Roos YB, Coutinho JM. Association between i.v. thrombolysis volume and door-to-needle times in acute ischemic stroke. J Neurol. 2016.											
Moran JL, Nakagawa K, Asai SM, Koenig MA. 24/7 Neurocritical Care Nurse Practitioner Coverage Reduced Door-to-Needle Time in Stroke Patients Treated with Tissue Plasminogen Activator. J Stroke Cerebrovasc Dis. 2016.		*			*			*		*	4
Rai AT, Smith MS, Boo S, Tarabishy AR, Hobbs GR, Carpenter JS. The ‘pit-crew’ model for improving door-to-needle times in endovascular stroke therapy: a Six-Sigma project. J Neurointerv Surg. 2016.	*	*	*			*		*	*	*	7
Choi PM, Desai JA, Kashyap D, Stephenson C, Kamal N, Vogt S, Bohm V, Suddes M, Bugbee E, Hill MD, Demchuk AM, Smith EE. Are All Stroke Patients Eligible for Fast Alteplase Treatment? An Analysis of Unavoidable Delays. Acad Emerg Med. 2016.	*	*		*	*		*	*	*		7
Sadeghi-Hokmabadi E, Taheraghdam A, Hashemilar M, Rikhtegar R, Mehrvar K, Mehrara M, Mirnour R, Hassasi R, Aliyar H, Farzi M, Hasaneh Tamar S. Simple In-Hospital Interventions to Reduce Door-to-CT Time in Acute Stroke. Int J Vasc Med. 2016.	*	*				*	*	*		*	6
Kim DH, Bae HJ, Han MK, Kim BJ, Park SS, Park TH, Lee KB, Kang K, Park JM, Ko Y, Lee SJ, Choi JC, Kim JT, Cho KH, Hong KS, Cho YJ, Kim DE, Lee J, Lee J, Oh MS, Yu KH, Lee BC, Nah HW, Cha JK. Direct admission to stroke centers reduces treatment delay and improves clinical outcome after intravenous thrombolysis. J Clin Neurosci. 2016.	*	*		*		*	*	*		*	7
Sim J, Shin CN, An K, Todd M. Factors Associated With the Hospital Arrival Time in Patients With Ischemic Stroke in Korea. J Cardiovasc Nurs. 2016.				*			*	*	*		4
Madsen TE, Sucharew H, Katz B, Alwell KA, Moomaw CJ, Kissela BM, Flaherty ML, Woo D, Khatri P, Ferioli S, Mackey J, Martini S, De Los Rios La Rosa F, Kleindorfer D. Gender and Time to Arrival among Ischemic Stroke Patients in the Greater Cincinnati/Northern Kentucky Stroke Study. J Stroke Cerebrovasc Dis. 2016.	*	*		*	*		*	*	*		7
Itrat A, Taqui A, Cerejo R, Briggs F, Cho SM, Organek N, Reimer AP, Winners S, Rasmussen P, Hussain MS, Uchino K; Cleveland Pre-Hospital Acute Stroke Treatment Group. Telemedicine in Prehospital Stroke Evaluation and Thrombolysis: Taking Stroke Treatment to the Doorstep. JAMA Neurol. 2016.	*	*	*	*	*		*	*	*	*	9
Busby L, Owada K, Dhungana S, Zimmermann S, Coppola V, Ruban R, Horn C, Rochestie D, Khaldi A, Hormes JT, Gupta R. CODE FAST: a quality improvement initiative to reduce door-to-needle times. J Neurointerv Surg. 2016.	*	*	*		*	*		*	*	*	8
Iglesias Mohedano AM, García Pastor A, García Arratibel A, Sobrino García P, Díaz Otero F, Romero Delgado F, Domínguez Rubio R, Muñoz González A, Vázquez Alen P, Fernández Bullido Y, Villanueva Osorio JA, Gil Núñez A. Factors associated with in-hospital delays in treating acute stroke with intravenous thrombolysis in a tertiary centre. Neurologia. 2016.		*		*	*		*	*	*		6
Van Schaik SM, Scott S, de Lau LM, Van den Berg-Vos RM, Kruyt ND. Short Door-to-Needle Times in Acute Ischemic Stroke and Prospective Identification of Its Delaying Factors. Cerebrovasc Dis Extra. 2015.	*	*		*	*		*	*	*		7
Sanossian N, Liebeskind DS, Eckstein M, Starkman S, Stratton S, Pratt FD, Koenig W, Hamilton S, Kim-Tenser M, Conwit R, Saver JL; FAST-MAG Investigators and Coordinators. Routing Ambulances to Designated Centers Increases Access to Stroke Center Care and Enrollment in Prehospital Research. Stroke. 2015.	*		*		*	*	*	*	*	*	8
Cerejo R, John S, Buletko AB, Taqui A, Itrat A, Organek N, Cho SM, Sheikhi L, Uchino K, Briggs F, Reimer AP, Winners S, Toth G, Rasmussen P, Hussain MS. A Mobile Stroke Treatment Unit for Field Triage of Patients for Intraarterial Revascularization Therapy. J Neuroimaging. 2015.	*	*		*	*	*		*	*	*	7
Chakraborty S, Ross J, Hogan MJ, Dowlatshahi D, Stotts G. Beating the clock: time delays to thrombolytic therapy with advanced imaging and impact of optimized workflow. J Stroke Cerebrovasc Dis. 2015.	*	*	*	*		*	*		*	*	8
Kim A, Lee JS, Kim JE, Paek YM, Chung K, Park JH, Cho YJ, Hong KS. Trends in yield of a code stroke program for enhancing thrombolysis. J Clin Neurosci. 2015.											
Wendt M, Ebinger M, Kunz A, Rozanski M, Waldschmidt C, Weber JE, Winter B, Koch PM, Freitag E, Reich J, Schremmer D, Audebert HJ; STEMO Consortium. Improved prehospital triage of patients with stroke in a specialized stroke ambulance: results of the pre-hospital acute neurological therapy and optimization of medical care in stroke study. Stroke. 2015.	*	*	*		*		*	*	*		7
Wolters FJ, Paul NL, Li L, Rothwell PM; Oxford Vascular Study. Sustained impact of UK FAST-test public education on response to stroke: a population-based time-series study. Int J Stroke. 2015.	*	*	*	*	*		*	*		*	8

* Blue ≥ 7 (good); orange: 5.6 (satisfactory); red ≤ 4 (unsatisfactory); ¥: pragmatic clinical trial.

**Table A3:** List of studies included in the systematic literature review.

Reference	Factors
Seo AR, Song H, Lee WJ, Park KN, Moon J, Kim D. Factors Associated with Delay of Emergency Medical Services Activation in Patients with Acute Stroke. J Stroke Cerebrovasc Dis. 2021 Jan; 30 (1): 105426.	Reduction of pre-hospital time (RPHT): activation and use of pre-hospital emergency; Reduction of in-hospital time (RIHT): pre-hospital notification
Kawano H, Ebisawa S, Ayano M, Kono Y, Saito M, Johno T, Maruoka H, Ryoji N, Yamashita H, Nakanishi K, Honda Y, Amano T, Unno Y, Komatsu Y, Ogawa Y, Shiokawa Y, Hirano T. Improving Acute In-Hospital Stroke Care by Reorganization of an In-Hospital Stroke Code Protocol. J Stroke Cerebrovasc Dis. 2021 Jan; 30 (1): 105433.	RIHT: development of the stroke protocol, training of all professionals in the hospital for stroke recognition (single stroke scales); use of posters in the hospital; publication in the newspaper/website of the institution of relevant information
Zhao H, Coote S, Easton D, Langenberg F, Stephenson M, Smith K, Bernard S, Cadilhac DA, Kim J, Bladin CF, Churilov L, Crompton DE, Dewey HM, Sanders LM, Wijeratne T, Cloud G, Brooks DM, Asadi H, Thijs V, Chandra RV, Ma H, Desmond PM, Dowling RJ, Mitchell PJ, Yassi N, Yan B, Campbell BCV, Parsons MW, Donnan GA, Davis SM. Melbourne Mobile Stroke Unit and Reperfusion Therapy: Greater Clinical Impact of Thrombectomy Than Thrombolysis. Stroke. 2020 Mar; 51 (3): 922-930.	RPHT and RIHT: ambulance with image and capacity for thrombolysis (urban area with high population density)
Ungerer MN, Busetto L, Begli NH, Riehle K, Regula J, Gumbinger C. Factors affecting prehospital delay in rural and urban patients with stroke: a prospective survey-based study in Southwest Germany. BMC Neurol. 2020 Dec 5; 20 (1): 441.	RPHT: activation and use of pre-hospital emergency
Le SM, Copeland LA, Zeber JE, Benge JF, Allen L, Cho J, Liao IC, Rasmussen J. Factors affecting time between symptom onset and emergency department arrival in stroke patients. eNeurologicalSci. 2020 Oct 24; 21: 100285	RIHT: pre-hospital notification
Nagao Y, Nakajima M, Inatomi Y, Ito Y, Kouzaki Y, Wada K, Yonehara T, Terasaki T, Hashimoto Y, Ando Y. Pre-Hospital Delay in Patients with Acute Ischemic Stroke in a Multicenter Stroke Registry: K-PLUS. J Stroke Cerebrovasc Dis. 2020 Nov; 29 (11): 105284.	RPHT: activation and use of pre-hospital emergency
Rynor H, Levine J, Souchak J, Shashoua N, Ramirez M, Gonzalez IC, Ramos V, Saxena A, Veledar E, Starosciak AK, Rios La Rosa FL. The Effect of a County Prehospital FAST-ED Initiative on Endovascular Treatment Times. J Stroke Cerebrovasc Dis. 2020 Nov; 29 (11): 105220.	RPHT: (thrombectomy): use of a specific clinical scale for recognizing proximal occlusion in the pre-hospital setting to directly refer the patient to a thrombectomy center; early direct notification to the neurologist (telemedicine) in the hospital and direct referral to the imaging room (cranioencephalic tomography); fibrinolysis in the imaging room.
Noone ML, Moideen F, Krishna RB, Pradeep Kumar VG, Karadan U, Chellenton J, Salam KA. Mobile App Based Strategy Improves Door-to-Needle Time in the Treatment of Acute Ischemic Stroke. J Stroke Cerebrovasc Dis. 2020 Dec; 29 (12): 105319.	RPHT: application for entering clinical data, time course of stroke, pre-hospital notification (shared in real time with the hospital)
Kielkopf M, Meinel T, Kaesmacher J, Fischer U, Arnold M, Heldner M, Seiffge D, Mordasini P, Dobrocky T, Piechowiak E, Gralla J, Jung S. Temporal Trends and Risk Factors for Delayed Hospital Admission in Suspected Stroke Patients. J Clin Med. 2020 Jul 25; 9 (8): 2376.	RPHT: activation and use of pre-hospital emergency
Xu H, Xian Y, Woon FP, Bettger JP, Laskowitz DT, Ng YY, Ong MEH, Matchar DB, De Silva DA. Emergency medical services use and its association with acute ischaemic stroke evaluation and treatment in Singapore. Stroke Vasc Neurol. 2020 Jun; 5 (2): 121-127.	RPHT: activation and use of pre-hospital emergency
Darehed D, Blom M, Glader EL, Niklasson J, Norrving B, Eriksson M. In-Hospital Delays in Stroke Thrombolysis: Every Minute Counts. Stroke. 2020 Aug; 51 (8): 2536-2539.	RIHT: direct admission for stoke unit, use of prehospital emergency
Miyamoto Y, Aso S, Iwagami M, Morita K, Fushimi K, Hamasaki Y, Nangaku M, Doi K, Yasunaga H. Expanded Indication for Recombinant Tissue Plasminogen Activator from 3 to 4.5 h after Onset of Stroke in Japan. J Stroke Cerebrovasc Dis. 2020 Dec; 29 (12): 105341.	RPHT: activation and use of pre-hospital emergency
Al Khathaami AM, Al Bdah B, Tarawneh M, Alskaini M, Alotaibi F, Alshalan A, Almuhraj M, Aldaham D, Alotaibi N In. Utilization of travenous Tissue Plasminogen Activator and Reasons for Nonuse in Acute Ischemic Stroke in Saudi Arabia. J Stroke Cerebrovasc Dis. 2020 May; 29 (5): 104761.	RPHT: activation and use of pre-hospital emergency
Zhu Y, Zhang X, You S, Cao X, Wang X, Gong W, Qin Y, Huang X, Cao Y, Shi R. Factors Associated with Pre-Hospital Delay and Intravenous Thrombolysis in China. J Stroke Cerebrovasc Dis. 2020 Aug; 29 (8): 104897.	RPHT: ambulance use
Mashni SK, O’Neal CR, Abner E, Lee J, Fraser JF. Time Intervals for Direct Versus Transfer Cases of Thrombectomy for Stroke in a Primarily Rural System of Care. J Stroke Cerebrovasc Dis. 2020 Jun; 29 (6): 104689. doi10.1016/j.jstrokecerebrovasdis.2020.104689. Epub 2020 Mar 6. PMID: 32151476; PMCID: PMC7246170.	RPHT: (thrombectomy): direct transfer to thrombectomy centers (versus to nearby center with thrombolysis) increases the number of patients benefiting from thrombectomy
Sloane B, Bosson N, Sanossian N, Saver JL, Perez L, Gausche-Hill M. Is Door-to-Needle Time Reduced for Emergency Medical Services Transported Stroke Patients Routed Directly to the Computed Tomography Scanner on Emergency Department Arrival? J Stroke Cerebrovasc Dis. 2020 Jan; 29 (1): 104477. doi: 10.1016/j.jstrokecerebrovasdis.2019.104477. Epub 2019 Nov 4. PMID: 31699573.	RIHT: direct admission to the imaging room and administration of fibrinolysis in the imaging room (no transfers to intermediate stretchers)
Sharma R, Zachrison KS, Viswanathan A, Matiello M, Estrada J, Anderson CD, Etherton M, Silverman S, Rost NS, Feske SK, Schwamm LH. Trends in Telestroke Care Delivery: A 15-Year Experience of an Academic Hub and Its Network of Spokes. Circ Cardiovasc Qual Outcomes. 2020 Mar; 13 (3): e005903.	RPHT: telestroke increased the number of patients treated with alteplase
Kummer BR, Lerario MP, Hunter MD, Wu X, Efraim ES, Salehi Omran S, Chen ML, Diaz IL, Sacchetti D, Lekic T, Kulick ER, Pishanidar S, Mir SA, Zhang Y, Asaeda G, Navi BB, Marshall RS, Fink ME. Geographic Analysis of Mobile Stroke Unit Treatment in a Dense Urban Area: The New York City METRONOME Registry. J Am Heart Assoc. 2019 Dec 17; 8 (24): e013529.	RPHT: “Mobile stroke unit” in a densely populated urban environment, the needle port time decreases.
Sanjuan Menéndez E, Girón Espot P, Calleja Macho L, Rodríguez-Samaniego MT, Santana Román KE, Rubiera Del Fueyo M. Implementation of a protocol for direct stroke patient transfer and mobilization of a stroke team to reduce times to reperfusion. Emergencias. 2019 Dic; 31 (6): 385-390. Spanish, English. PMID: 31777209.	RIHT: direct admission to the imaging room and administration of fibrinolysis in the imaging room (no transfers to intermediate stretchers)
Soto-Cámara R, González-Santos J, González-Bernal J, Martín-Santidrian A, Cubo E, Trejo-Gabriel-Galán JM. Factors Associated with Shortening of Prehospital Delay among Patients with Acute Ischemic Stroke. J Clin Med. 2019 Oct 17; 8 (10): 1712.	RPHT: ambulance use and pre-hospital notification
Madhok DY, Keenan KJ, Cole SB, Martin C, Hemphill JC 3rd. Prehospital and Emergency Department-Focused Mission Protocol Improves Thrombolysis Metrics for Suspected Acute Stroke Patients. J Stroke Cerebrovasc Dis. 2019 Dec; 28 (12): 104423.	RPHT and RIHT: implementation of a protocol for the pre-hospital emergency and for the urgency (up to the imaging room)
Fladt J, Meier N, Thilemann S, Polymeris A, Traenka C, Seiffge DJ, Sutter R, Peters N, Gensicke H, Flückiger B, de Hoogh K, Künzli N, Bringolf-Isler B, Bonati LH, Engelter ST, Lyrer PA, De Marchis GM. Reasons for Prehospital Delay in Acute Ischemic Stroke. J Am Heart Assoc. 2019 Oct 15; 8 (20): e013101.	RPHT: ambulance use and pre-hospital notification
Bonadio W, Beck C, Mueller A. Impact of CT scanner location on door to imaging time for emergency department stroke evaluation. Am J Emerg Med. 2020 Feb; 38 (2): 309-310.	RIHT: placement of the imaging room near the emergency room
Choi PMC, Tsoi AH, Pope AL, Leung S, Frost T, Loh PS, Chandra RV, Ma H, Parsons M, Mitchell P, Dewey HM. Door-in-Door-Out Time of 60 Minutes for Stroke With Emergent Large Vessel Occlusion at a Primary Stroke Center. Stroke. 2019 Oct; 50 (10): 2829-2834.	RIHT: (thrombectomy): protocol and audit for identification/correction of the entire chain of in-hospital actors (nursing, analysis and imaging) in stroke
Jagolino-Cole AL, Bozorgui S, Ankrom CM, Vahidy F, Bambhroliya AB, Randhawa J, Trevino AD, Cossey TC, Savitz SI, Wu TC. Variability and Delay in Telestroke Physician Alert among Spokes in a Telestroke Network: A Need for Metric Benchmarks. J Stroke Cerebrovasc Dis. 2019 Nov; 28 (11): 104332.	In-hospital time delay (IHTD): wait for brain image result before activating telestroke query
Dimitriou P, Tziomalos K, Christou K, Kostaki S, Angelopoulou SM, Papagianni M, Ztriva E, Chatzopoulos G, Savopoulos C, Hatzitolios AI. Factors associated with delayed presentation at the emergency department in patients with acute ischemic stroke. Brain Inj. 2019; 33 (9): 1257-1261.	RPHT: ambulance use and pre-hospital notification
Nepal G, Yadav JK, Basnet B, Shrestha TM, Kharel G, Ojha R. Status of prehospital delay and intravenous thrombolysis in the management of acute ischemic stroke in Nepal. BMC Neurol. 2019 Jul 9; 19 (1): 155.	RPHT: knowledge of the existence of a stroke treatment; ambulance use and pre-hospital notification
Gonzalez-Aquines A, Cordero-Pérez AC, Cristobal-Niño M, Pérez-Vázquez G, Góngora-Rivera F; GECEN Investigators. Contribution of Onset-to-Alarm Time to Prehospital Delay in Patients with Ischemic Stroke. J Stroke Cerebrovasc Dis. 2019 Nov; 28 (11): 104331.	RPHT: ambulance use and pre-hospital notification
Bhaskar S, Thomas P, Cheng Q, Clement N, McDougall A, Hodgkinson S, Cordato D. Trends in acute stroke presentations to an emergency department: implications for specific communities in accessing acute stroke care services. Postgrad Med J. 2019 May; 95 (1123): 258-264.	RPHT: ambulance use and pre-hospital notification
Chai E, Li C, Jiang L. Factors affecting in-hospital delay of intravenous thrombolysis for acute ischemic stroke: A retrospective cohort study. Medicine (Baltimore). 2019 May; 98 (19): e15422	RPHT: ambulance use and pre-hospital notification
Weisenburger-Lile D, Blanc R, Kyheng M, Desilles JP, Labreuche J, Piotin M, Mazighi M, Consoli A, Lapergue B, Gory B; on behalf of the Endovascular Treatment in Ischemic Stroke Investigators. Direct Admission versus Secondary Transfer for Acute Stroke Patients Treated with Intravenous Thrombolysis and Thrombectomy: Insights from the Endovascular Treatment in Ischemic Stroke Registry. Cerebrovasc Dis. 2019; 47 (3-4): 112-120.	RPHT: (thrombectomy): direct transfer to thrombectomy centers (versus to nearby center with thrombolysis) increases the number of patients benefiting from thrombectomy
Varjoranta T, Raatiniemi L, Majamaa K, Martikainen M, Liisanantti JH. Prehospital and hospital delays for stroke patients treated with thrombolysis: A retrospective study from mixed rural-urban area in Northern Finland. Australas Emerg Care. 2019 Jun; 22 (2): 76-80.	RPHT: stroke assessment protocols; use of ambulance and pre-hospital notification
Han TS, Gulli G, Affley B, Fluck D, Fry CH, Barrett C, Kakar P, Sharma S, Sharma P. New evidence-based A1, A2, A3 alarm time zones for transferring thrombolysed patients to hyper-acute stroke units: faster is better. Neurol Sci. 2019 Aug; 40 (8): 1659-1665.	RPHT: division of the area of influence of each hospital by different priority zones for prioritizing the means.
Manners J, Khandker N, Barron A, Aziz Y, Desai SM, Morrow B, Delfyett WT, Martin-Gill C, Shutter L, Jovin TG, Jadhav AP. An interdisciplinary approach to inhospital stroke improves stroke detection and treatment time. J Neurointerv Surg. 2019 Nov; 11 (11): 1080-1084.	RIHT: Instituting a Stroke Protocol improves care provided in thrombolysis and thrombectomy
Nordanstig A, Palaszewski B, Asplund K, Norrving B, Wahlgren N, Wester P, Jood K, Rosengren L. Evaluation of the Swedish National Stroke Campaign: A population-based time-series study. Int J Stroke. 2019 Dec; 14 (9): 862-870.	RPHT: national campaign to raise awareness of stroke and its treatment reduced pre-hospital time and increased the rate of thrombolysis.
Gu HQ, Rao ZZ, Yang X, Wang CJ, Zhao XQ, Wang YL, Liu LP, Wang CY, Liu C, Li H, Li ZX, Xiao RP, Wang YJ; Chinese Stroke Center Alliance Investigators. Use of Emergency Medical Services and Timely Treatment Among Ischemic Stroke. Stroke. 2019 Apr; 50 (4): 1013-1016.	RPHT and RIHT: ambulance use and pre-hospital notification
Faiz KW, Sundseth A, Thommessen B, Rønning OM. The knowing-doing gap in acute stroke-Does stroke knowledge translate into action? Brain Behav. 2019 Mar; 9 (3): e01245.	RPHT: first contact for pre-hospital emergency number
Jung S, Rosini JM, Nomura JT, Caplan RJ, Raser-Schramm J. Even Faster Door-to-Alteplase Times and Associated Outcomes in Acute Ischemic Stroke. J Stroke Cerebrovasc Dis. 2019 Dec; 28 (12): 104329.	RIHT: “Comprehensive Stroke Center” (compared to primary stroke centers)—implies high-volume, better-differentiated centers versus low-volume, less-differentiated centers.
Sobral S, Taveira I, Seixas R, Vicente AC, Duarte J, Goes AT, Durán D, Lopes J, Rita H, Nzwalo H. Late Hospital Arrival for Thrombolysis after Stroke in Southern Portugal: Who Is at Risk? J Stroke Cerebrovasc Dis. 2019 Apr; 28 (4): 900-905.	RPHT: use of ambulance as a way to get to the hospital
Klingner C, Günther A, Brodoehl S, Witte OW, Klingner CM. Talk About Thrombolysis. Regular Case-Based Discussions of Stroke Thrombolysis Improve Door-to-Needle Time by 20. J Stroke Cerebrovasc Dis. 2019 Apr; 28 (4): 876-881.	RIHT: regular discussion of clinical cases related to the green pathway of stroke combined with discussion.
Kamal N, Shand E, Swanson R, Hill MD, Jeerakathil T, Imoukhuede O, Heinrichs I, Bakker J, Stoyberg C, Fowler L, Duckett S, Holsworth S, Mann B, Valaire S, Bestard J. Reducing Door-to-Needle Times for Ischaemic Stroke to a Median of 30 Minutes at a Community Hospital. Can J Neurol Sci. 2019 Jan; 46 (1): 51-56.	RIHT: pre-hospital notification, physician from the Stoke unit to the emergency room, direct referral to the imaging room and thrombolysis in the imaging room
Mourand I, Malissart P, Dargazanli C, Nogue E, Bouly S, Gaillard N, Boukriche Y, Corti L, Picot MC, Beaufils O, Chbicheb M, Sablot D, Bonafe A, Costalat V, Arquizan C. A Regional Network Organization for Thrombectomy for Acute Ischemic Stroke in the Anterior Circulation; Timing, Safety, and Effectiveness. J Stroke Cerebrovasc Dis. 2019 Feb; 28 (2): 259-266.	RPHT: (thrombectomy): protocol to identify patients with a high probability of having proximal occlusion and refer directly to a thrombectomy center
Menon BK, Xu H, Cox M, Saver JL, Goyal M, Peterson E, Xian Y, Matsuoka R, Jehan R, Yavagal D, Gupta R, Mehta B, Bhatt DL, Fonarow GC, Schwamm LH, Smith EE. Components and Trends in Door to Treatment Times for Endovascular Therapy in Get With The Guidelines-Stroke Hospitals. Circulation. 2019 Jan 8; 139 (2): 169-179.	RPHT and RIHT (thrombectomy): use of ambulance and pre-hospital notification; direct referral to thrombectomy centers in patients with suspected proximal lesion
Heikkilä I, Kuusisto H, Holmberg M, Palomäki A. Fast Protocol for Treating Acute Ischemic Stroke by Emergency Physicians. Ann Emerg Med. 2019 Feb; 73 (2): 105-112.	RIHT: implementation of a mandatory “FAST” protocol after education of emergency service personnel.
Hassankhani H, Soheili A, Vahdati SS, Mozaffari FA, Fraser JF, Gilani N. Treatment Delays for Patients With Acute Ischemic Stroke in an Iranian Emergency Department: A Retrospective Chart Review. Ann Emerg Med. 2019 Feb; 73 (2): 118-129.	RPHT: use of ambulance as a way to get to the hospital IHTD: call the stoke unit doctor after performing the imaging exam
Olascoaga Arrate A, Freijo Guerrero MM, Fernández Maiztegi C, Azkune Calle I, Silvariño Fernández R, Fernández Rodríguez M, Vazquez Naveira P, Anievas Elena A, Iturraspe González I, Pérez Díez Y, Ruiz Fernández R. Use of emergency medical transport and impact on time to care in patients with ischaemic stroke. Neurologia. 2019 Mar; 34 (2): 80-88.	RPHT and RIHT: ambulance use and pre-hospital notification
Tong X, Wiltz JL, George MG, Odom EC, Coleman King SM, Chang T, Yin X; Paul Coverdell National Acute Stroke Program team, Merritt RK. A Decade of Improvement in Door-to-Needle Time Among Acute Ischemic Stroke Patients, 2008 to 2017. Circ Cardiovasc Qual Outcomes. 2018 Dec; 11 (12): e004981.	RIHT: implementation program for adherence to ischemic stroke guidelines
Hebant B, Triquenot-Bagan A, Guegan-Massardier E, Ozkul-Wermester O, Maltête D. In-hospital delays to stroke thrombolysis: Out of hours versus regular hours and reduction in treatment times through the creation of a 24/7 mobile thrombolysis team. J Neurol Sci. 2018 Sep 15; 392: 46-50.	RPHT: “mobile stroke unit” or ambulance with thrombolysis
Cone DC, Cooley C, Ferguson J, Harrell AJ, Luk JH, Martin-Gill C, Marquis SW, Pasichow S. Observational Multicenter Study of a Direct-to-CT Protocol for EMS-transported Patients with Suspected Stroke. Prehosp Emerg Care. 2018 Jan-Feb; 22 (1): 1-6.	RIHT: pre-notification and direct patient transport to the imaging room
Al Khathaami AM, Mohammad YO, Alibrahim FS, Jradi HA. Factors associated with late arrival of acute stroke patients to emergency department in Saudi Arabia. *SAGE Open Med*. 2018; 6: 2050312118776719.	RPHT: use of ambulance as a way to get to the hospital
Moreno A., Schwamm LH, Siddiqui KA, Viswanathan A, Whitney C, Rost N, Zachrison KS. Frequent Hub-Spoke Contact Is Associated with Improved Spoke Hospital Performance: Results from the Massachusetts General Hospital Telestroke Network. Telemed J E Health. 2018 Sep; 24 (9): 678-683.	RIHT: telestroke network between the central hospital and the peripheral.
Gonzalez-Aquines A, Cordero-Perez AC, Ramirez-Martinez LA, Sanchez-Teran H, Escobedo-Zuñiga N, Treviño-Herrera AB, Gongora-Rivera F, Pérez-Vázquez G, Contreras-Salazar K, Gómez-Padilla D. Onset-to-alarm time in patients with acute stroke: Results from a Mexican population. Int J Stroke. 2018 Oct; 13 (7): NP19-NP21.	RPHT: use of ambulance as a way to get to the hospital
Man S, Zhao X, Uchino K, Hussain MS, Smith EE, Bhatt DL, Xian Y, Schwamm LH, Shah S, Khan Y, Fonarow GC. Comparison of Acute Ischemic Stroke Care and Outcomes Between Comprehensive Stroke Centers and Primary Stroke Centers in the United States. Circ Cardiovasc Qual Outcomes. 2018 Jun; 11 (6): e004512.	RPHT: use of ambulance as a way to get to the hospital
Bohmann FO, Tahtali D, Kurka N, Wagner M, You SJ, du Mesnil de Rochemont R, Berkefeld J, Hartmetz AK, Kuhlmann A, Lorenz MW, Schütz A, Kress B, Henke C, Tritt S, Meyding-Lamadé U, Steinmetz H, Pfeilschifter W. A Network-Wide Stroke Team Program Reduces Time to Treatment for Endovascular Stroke Therapy in a Regional Stroke-Network. Cerebrovasc Dis. 2018; 45 (3-4): 141-148.	RIHT: comprehensive training of cognitive and non-cognitive skills to accelerate
Springer MV, Labovitz DL. The Effect of Being Found with Stroke Symptoms on Predictors of Hospital Arrival. J Stroke Cerebrovasc Dis. 2018 May; 27 (5): 1363-1367.	RPHT: use of ambulance as a way to get to the hospital
Andersson Hagiwara M, Wireklint Sundström B, Brink P, Herlitz J, Hansson PO. A shorter system delay for haemorrhagic stroke than ischaemic stroke among patients who use emergency medical service. Acta Neurol Scand. 2018 May; 137 (5): 523-530.	RPHT: use of ambulance as a way to get to the hospital
Tan BYQ, Ngiam NJH, Sunny S, Kong WY, Tam H, Sim TB, Leong BSH, Bhartendu C, Paliwal PR, Seet RCS, Chan BPL, Teoh HL, Sharma VK, Yeo LLL. Improvement in Door-to-Needle Time in Patients with Acute Ischemic Stroke via a Simple Stroke Activation Protocol. J Stroke Cerebrovasc Dis. 2018 Jun; 27 (6): 1539-1545.	RIHT: stroke chronometer and simple stroke activation protocol
Haesebaert J, Nighoghossian N, Mercier C, Termoz A, Porthault S, Derex L, Gueugniaud PY, Bravant E, Rabilloud M, Schott AM; AVC II Trial group. Improving Access to Thrombolysis and Inhospital Management Times in Ischemic Stroke: A Stepped-Wedge Randomized Trial. Stroke. 2018 Feb; 49 (2): 405-411.	RIHT: protocol and audit to identify/correct the entire chain of in-hospital stroke participants; provision of educational material (video, articles, posters).
Nguyen-Huynh MN, Klingman JG, Avins AL, Rao VA, Eaton A, Bhopale S, Kim AC, Morehouse JW, Flint AC; KPNC Stroke FORCE Team. Novel Telestroke Program Improves Thrombolysis for Acute Stroke Across 21 Hospitals of an Integrated Healthcare System. Stroke. 2018 Jan; 49 (1): 133-139.	RIHT: *telestroke* and application of the *Helsinki* standard protocol
Hsieh MJ, Chien KL, Sun JT, Tang SC, Tsai LK, Chiang WC, Chien YC, Jeng JS, Huei-Ming Ma M; Taipei EMS Stroke Collaborative Group. The effect and associated factors of dispatcher recognition of stroke: A retrospective observational study. J Formos Med Assoc. 2018 Oct; 117 (10): 902-908.	Pre-hospital time delay (PHTD): violation of assessment protocols and computerization by pre-hospital professionals
García Ruiz R, Silva Fernández J, García Ruiz RM, Recio Bermejo M, Arias Arias Á, Del Saz Saucedo P, Huertas Arroyo R, González Manero A, Santos Pinto A, Navarro Muñoz S, Botia Paniagua E, Abellán Alemán J. Response to Symptoms and Prehospital Delay in Stroke Patients. Is It Time to Reconsider Stroke Awareness Campaigns? J Stroke Cerebrovasc Dis. 2018 Mar; 27 (3): 625-632.	RPHT and RIHT: ambulance use and pre-hospital notification
Al Kasab S, Harvey JB, Debenham E, Jones DJ, Turner N, Holmstedt CA. Door to Needle Time over Telestroke-A Comprehensive Stroke Center Experience. Telemed J E Health. 2018 Feb; 24 (2): 111-115.	RIHT: education program, promoting quality and increasing confidence in stroke care
Lawrence E, Merbach D, Thorpe S, Llinas RH, Marsh EB. Streamlining the Process for Intravenous Tissue Plasminogen Activator. J Neurosci Nurs. 2018 Feb; 50 (1): 37-41.	RIHT: introduction of a flowchart guided by nurses
Denis Sablot, Ioana Ion, Khaled Khlifa, Geoffroy Farouil, Franck Leibinger, Nicolas Gaillard, Alexandre Laverdure, Zoubir Mourad Bensalah, Julie Mas, Bénédicte Fadat, Philippe Smadja, Adélaïde Ferraro-Allou, Jean-Marie Bonnec, Nadège Olivier, Anaïs Dutray~, Maxime Tardieu, Adrian Dumitrana, Aymeric Guibal, Snejana Jurici, Jean-Louis Bertrand, Thibaut Allou, Caroline Arquizan, Alain Bonafe. Target Door-to-Needle Time for Tissue Plasminogen Activator Treatment with Magnetic ResonanceImaging Screening Can Be Reduced to 45 min. Cerebrovasc Dis 2018; 45: 245–251.	RIHT: replacement of cranial tomography by cranial magnetic resonance; administration of thrombolysis in the imaging room.
Brown CW, Macleod MJ. The positive predictive value of an ambulance prealert for stroke and transient ischaemic attack. Eur J Emerg Med. 2018 Dec; 25 (6): 411-415.	RIHT: training of paramedical personnel for pre-hospital identification and notification
Ribo M, Boned S, Rubiera M, Tomasello A, Coscojuela P, Hernández D, Pagola J, Juega J, Rodriguez N, Muchada M, Rodriguez-Luna D, Molina CA. Direct transfer to angiosuite to reduce door-to-puncture time in thrombectomy for acute stroke. J Neurointerv Surg. 2018 Mar; 10 (3): 221-224.	RIHT (thrombectomy): direct transfer of the patient to the angiography room
Abraham SV, Krishnan SV, Thaha F, Balakrishnan JM, Thomas T, Palatty BU. Factors delaying management of acute stroke: An Indian scenario. Int J Crit Illn Inj Sci. 2017 Oct-Dec; 7 (4): 224-230.	PHTD: absence of specific protocols for stroke IHTD: distance between the emergency department and the imaging room
Springer MV, Labovitz DL, Hochheiser EC. Race-Ethnic Disparities in Hospital Arrival Time after Ischemic Stroke. Ethn Dis. 2017 Apr 20; 27 (2): 125-132.	RPHT: use of ambulance as a way to get to the hospital
Denti L, Caminiti C, Scoditti U, Zini A, Malferrari G, Zedde ML, Guidetti D, Baratti M, Vaghi L, Montanari E, Marcomini B, Riva S, Iezzi E, Castellini P, Olivato S, Barbi F, Perticaroli E, Monaco D, Iafelice I, Bigliardi G, Vandelli L, Guareschi A, Artoni A, Zanferrari C, Schulz PJ. Impact on Prehospital Delay of a Stroke Preparedness Campaign: A SW-RCT (Stepped-Wedge Cluster Randomized Controlled Trial). Stroke. 2017 Dec; 48 (12): 3316-3322.	Negative result: campaign for the population did not result in a reduction in pre-hospital time
Bray JE, Denisenko S, Campbell BCV, Stephenson M, Muller J, Hocking G, Hand PJ, Bladin CF. Strategic framework improves access to stroke reperfusion across the state of Victoria Australia. Intern Med J. 2017 Aug; 47 (8): 923-928.	RPHT and RIHT: strategic and specific for a region, taking into account the distances of population clusters in relation to stroke units (redistribution of ambulances and pre-hospital referrals)
Iglesias Mohedano AM, García Pastor A, Díaz Otero F, Vázquez Alen P, Vales Montero M, Luque Buzo E, Redondo Ráfales N, Chavarria Cano B, Fernández Bullido Y, Villanueva Osorio JA, Gil Núñez A. Efficacy of New Measures Saving Time in Acute Stroke Management: A Quantified Analysis. J Stroke Cerebrovasc Dis. 2017 Aug; 26 (8): 1817-1823.	RIHT: pre-hospital notification; elimination of all tests/interventions that are not essential for thrombolysis (hemostasis study, electrocardiogram); request for cranioencephalic tomography before the patient arrives at the hospital
Andrew BY, Stack CM, Yang JP, Dodds JA. mStroke: “Mobile Stroke”-Improving Acute Stroke Care with Smartphone Technology. J Stroke Cerebrovasc Dis. 2017 Jul; 26 (7): 1449-1456.	RIHT: computer application that allows the coordination of pre-hospital and hospital care in real time
Mowla A, Doyle J, Lail NS, Rajabzadeh-Oghaz H, Deline C, Shirani P, Ching M, Crumlish A, Steck DA, Janicke D, Levy EI, Sawyer RN. Delays in door-to-needle time for acute ischemic stroke in the emergency department: A comprehensive stroke center experience. J Neurol Sci. 2017 May 15; 376: 102-105.	IHTD: ineffective screening protocols, image acquisition and interpretation
Wireklint Sundström B, Andersson Hagiwara M, Brink P, Herlitz J, Hansson PO. The early chain of care and risk of death in acute stroke in relation to the priority given at the dispatch centre: A multicentre observational study. Eur J Cardiovasc Nurs. 2017 Oct; 16 (7): 623-631.	IHTD: low priority assignment by prehospital emergency personnel
Eriksson M, Glader EL, Norrving B, Stegmayr B, Asplund K. Acute stroke alert activation, emergency service use, and reperfusion therapy in Sweden. Brain Behav. 2017 Mar 15; 7 (4): e00654.	RIHT: pre-hospital notification
Hillen ME, He W, Al-Qudah Z, Wang W, Hidalgo A, Walia J. Long-Term Impact of Implementation of a Stroke Protocol on Door-to-Needle Time in the Administration of Intravenous Tissue Plasminogen Activator. J Stroke Cerebrovasc Dis. 2017 Jul; 26 (7): 1569-1572.	RIHT: direct forwarding to the imaging room; continuous monitoring of in-hospital stroke greenway times
Threlkeld ZD, Kozak B, McCoy D, Cole S, Martin C, Singh V. Collaborative Interventions Reduce Time-to-Thrombolysis for Acute Ischemic Stroke in a Public Safety Net Hospital. J Stroke Cerebrovasc Dis. 2017 Jul; 26 (7): 1500-1505.	RIHT: pre-notification; “CVA medication box” (antihypertensive drugs, equipment), protocol for pharmacy/imaging room notification; time reduction for image report
Liu Q, Ranta AA, Abernethy G, Barber PA. Trends in New Zealand stroke thrombolysis treatment rates. N Z Med J. 2017 Apr 7; 130 (1453): 50-56.	RPHT: National registry of patients undergoing thrombolysis combined with: telestroke in hospitals without access to stroke specialists; stroke recognition promotion campaign (“FAST”); improved coordination of pre-hospital emergency services; pre-notification education by ambulance services; RIHT: direct forwarding to the imaging room; administration of thrombolysis in the emergency department
Taqui A, Cerejo R, Itrat A, Briggs FB, Reimer AP, Winners S, Organek N, Buletko AB, Sheikhi L, Cho SM, Buttrick M, Donohue MM, Khawaja Z, Wisco D, Frontera JA, Russman AN, Hustey FM, Kralovic DM, Rasmussen P, Uchino K, Hussain MS; Cleveland Pre-Hospital Acute Stroke Treatment (PHAST) Group. Reduction in time to treatment in prehospital telemedicine evaluation and thrombolysis. Neurology. 2017 Apr 4; 88 (14): 1305-1312.	RPHT and RIHT: the introduction of a telemedicine model with thrombolysis performed in an ambulance equipped with cranial tomography
Khurana D, Das B, Kumar A, Kumar S A, Khandelwal N, Lal V, Prabhakar S. Temporal Trends in Intravenous Thrombolysis in Acute Ischemic Stroke: Experience from a Tertiary Care Center in India. J Stroke Cerebrovasc Dis. 2017 Jun; 26 (6): 1266-1273.	RPHT: ambulance use
Kamal N, Sheng S, Xian Y, Matsouaka R, Hill MD, Bhatt DL, Saver JL, Reeves MJ, Fonarow GC, Schwamm LH, Smith EE. Delays in Door-to-Needle Times and Their Impact on Treatment Time and Outcomes in Get With The Guidelines-Stroke. Stroke. 2017 Apr; 48 (4): 946-954.	RPHT: ambulance use/pre-hospital notification IHTD: constraints related to access to equipment (image room, monitoring/analysis devices)
Martin A. Reznek, MD, MBA; Evangelia Murray, MS; Marguerite N. Youngren, BA; Natassia T. Durham, MSIE; Sean S. Michael, MD. Door-to-Imaging Time for Acute Stroke Patients Is Adversely Affected by Emergency Department Crowding. Stroke. 2017; 48: 00-00.	RPHT: ambulance use
Sablot D, Gaillard N, Colas C, Smadja P, Gely C, Dutray A, Bonnec JM, Jurici S, Farouil G, Ferraro-Allou A, Jantac M, Allou T, Pujol C, Olivier N, Laverdure A, Fadat B, Mas J, Dumitrana A, Garcia Y, Touzani H, Perucho P, Moulin T, Richard C, Heroum C, Bouly S, Sagnes-Raffy C, Heve D. Results of a 1-year quality-improvement process to reduce door-to-needle time in acute ischemic stroke with MRI screening. Rev Neurol (Paris). 2017 Jan-Feb; 173 (1-2): 47-54.	RIHT protocol: pre-hospital notification, replacement of cranial tomography with cranial magnetic resonance; administration of thrombolysis in the imaging room.
Slivinski A, Jones R, Whitehead H, Hooper V. Improving Access to Stroke Care in the Rural Setting: The Journey to Acute Stroke Ready Designation. J Emerg Nurs. 2017 Jan; 43 (1): 24-32.	RPHT and RIHT: care protocol developed by a multidisciplinary team dominated by professionals from the emergency department, but also involving the community: telemedicine, pre-notification, flowchart of patients with stroke
Kamal N, Holodinsky JK, Stephenson C, Kashayp D, Demchuk AM, Hill MD, Vilneff RL, Bugbee E, Zerna C, Newcommon N, Lang E, Knox D, Smith EE. Improving Door-to-Needle Times for Acute Ischemic Stroke: Effect of Rapid Patient Registration, Moving Directly to Computed Tomography, and Giving Alteplase at the Computed Tomography Scanner. Circ Cardiovasc Qual Outcomes. 2017 Jan; 10 (1): e003242.	RIHT: rapid patient registration on admission, thrombolysis bag, direct transport to the imaging room, thrombolysis in the imaging room
Xian Y, Xu H, Lytle B, Blevins J, Peterson ED, Hernandez AF, Smith EE, Saver JL, Messé SR, Paulsen M, Suter RE, Reeves MJ, Jauch EC, Schwamm LH, Fonarow GC. Use of Strategies to Improve Door-to-Needle Times With Tissue-Type Plasminogen Activator in Acute Ischemic Stroke in Clinical Practice: Findings from Target: Stroke.Circ Cardiovasc Qual Outcomes. 2017 Jan; 10 (1): e003227.	RIHT: pre-notification, rapid screening, specific stroke protocol, thrombolysis in the imaging room There is a need to monitor these parameters
Puy L, Lamy C, Canaple S, Arnoux A, Laine N, Iacob E, Constans JM, Godefroy O. Creation of an intensive care unit and organizational changes in an adult emergency department: Impact on acute stroke management. Am J Emerg Med. 2017 May; 35 (5): 716-719.	RIHT: creation of an intensive care unit, within the Medical Emergency department, where stroke patients are also treated.
Chen BY, Moussaddy A, Keezer MR, Deschaintre Y, Poppe AY. Short- and Long-Term Reduction of Door-to-Needle Time in Thrombolysis for Acute Stroke. Can J Neurol Sci. 2017 May; 44 (3): 255-260.	RPHT: implementation of the Helsinki model (pre-notification, direct transfer to the imaging room, thrombolysis in the imaging room)
Reznek MA, Murray E, Youngren MN, Durham NT, Michael SS. Door-to-Imaging Time for Acute Stroke Patients Is Adversely Affected by Emergency Department Crowding. Stroke. 2017 Jan; 48 (1): 49-54.	RPHT: ambulance use IHTD: equipment constraints (“overcrowding of imaging rooms”)
Metts EL, Bailey AM, Weant KA, Justice SB. Identification of Rate-Limiting Steps in the Provision of Thrombolytics for Acute Ischemic Stroke. J Pharm Pract. 2017 Dec; 30 (6): 606-611.	IHTD: immediate evaluation in the emergency room, absence of free access to the imaging room and for the analyzes
Zhou Y, Yang T, Gong Y, Li W, Chen Y, Li J, Wang M, Yin X, Hu B, Lu Z. Pre-hospital Delay after Acute Ischemic Stroke in Central Urban China: Prevalence and Risk Factors. Mol Neurobiol. 2017 May; 54 (4): 3007-3016.	RPHT: use of the national emergency number
Aghaebrahim A, Streib C, Rangaraju S, Kenmuir CL, Giurgiutiu DV, Horev A, Saeed Y, Callaway CW, Guyette FX, Martin-Gill C, Pacella C, Ducruet AF, Jankowitz BT, Jovin TG, Jadhav AP. Streamlining door to recanalization processes in endovascular stroke therapy. J Neurointerv Surg. 2017 Apr; 9 (4): 340-345.	RIHT (thrombectomy): implementation of stroke code: pre-notification, direct referral to imaging room, thrombolysis bag, administration in imaging room; eliminate all redundant exams; in cases of thrombectomy: pre-notification and referral to the thrombectomy room
Siju V. Abraham, S. Vimal Krishnan, Fazil Thaha, Jayaraj Mymbilly Balakrishnan, Tom Thomas, and Babu Urumese Palatty. Factors delaying management of acute stroke: An Indian scenario. Int J Crit Illn Inj Sci. 2017 Oct-Dec; 7 (4): 224–230.	IHTD: absence of ambulances; distance between the Stroke Unit and the imaging room
Zinkstok SM, Beenen LF, Luitse JS, Majoie CB, Nederkoorn PJ, Roos YB. Thrombolysis in Stroke within 30 Minutes: Results of the Acute Brain Care Intervention Study. PLoS One. 2016 Nov 18; 11 (11): e0166668.	RPHT: program for education, pre-notification, exclusion of contraindications while still in the ambulance RIHT: pre-registration of patients, direct referral to the imaging room, stretchers with patient weighing
Hubert GJ, Meretoja A, Audebert HJ, Tatlisumak T, Zeman F, Boy S, Haberl RL, Kaste M, Müller-Barna P. Stroke Thrombolysis in a Centralized and a Decentralized System (Helsinki and Telemedical Project for Integrative Stroke Care Network). Stroke. 2016 Dec; 47 (12): 2999-3004.	RPHT and RIHT: model based on telemedicine in rural areas is superimposed on the Helsinki model
Prabhakaran S, Lee J, O’Neill K. Regional Learning Collaboratives Produce Rapid and Sustainable Improvements in Stroke Thrombolysis Times. Circ Cardiovasc Qual Outcomes. 2016 Sep; 9 (5): 585-92.	RIHT: implementation of a multicenter program, with multidisciplinary discussions and continuous monitoring: pre-notification, direct referral to the imaging room, thrombolysis bag, administration in the imaging room
Belt GH, Felberg RA, Rubin J, Halperin JJ. In-Transit Telemedicine Speeds Ischemic Stroke Treatment: Preliminary Results. Stroke. 2016 Sep; 47 (9): 2413-5.	RPHT and RIHT: ambulance with telemedicine-guided imaging and thrombolysis in densely populated urban spaces It is cost-effective only during the day.
Park MS, Lee JS, Park TH, Cho YJ, Hong KS, Park JM, Kang K, Lee KB, Kim JG, Lee SJ, Lee J, Choi KH, Kim JT, Cho KH, Oh MS, Yu KH, Lee BC, Cha JK, Kim DH, Nah HW, Lee J, Kim DE, Ryu WS, Kim BJ, Han MK, Bae HJ, Song SK, Choi JC. Characteristics of the Drip-and-Ship Paradigm for Patients with Acute Ischemic Stroke in South Korea. J Stroke Cerebrovasc Dis. 2016 Nov; 25 (11): 2678-2687.	PHTD and IHTD (thrombectomy): “drip and ship” model as opposed to the motherboard model
Birnbaum LA, Rodriguez JS, Topel CH, Behrouz R, Misra V, Palacio S, Patterson MG, Motz DS, Goros MW, Cornell JE, Caron JR. Older Stroke Patients with High Stroke Scores Have Delayed Door-To-Needle Times. J Stroke Cerebrovasc Dis. 2016 Nov; 25 (11): 2668-2672.	RPHT: ambulance use
Marto JP, Borbinha C, Calado S, Viana-Baptista M. The Stroke Chronometer-A New Strategy to Reduce Door-to-Needle Time. J Stroke Cerebrovasc Dis. 2016 Sep; 25 (9): 2305-7.	RIHT: use of a “stroke timer” from patient entry to fibrinolysis
Koch PM, Kunz A, Ebinger M, Geisler F, Rozanski M, Waldschmidt C, Weber JE, Wendt M, Winter B, Zieschang K, Bollweg K, Kaczmarek S, Endres M, Audebert HJ. Influence of Distance to Scene on Time to Thrombolysis in a Specialized Stroke Ambulance. Stroke. 2016 Aug; 47 (8): 2136-40.	RPHT and RIHT: ambulance with telemedicine-guided imaging and thrombolysis in patients located at a temporal distance greater than 18 min.
Ibrahim F, Akhtar N, Salam A, Kamran S, Deleu D, D’Souza A, Imam Y, Bourke P, Joseph S, Santos M, Khan R, Bhutta ZA, Bhagat A, Shuaib A. Stroke Thrombolysis Protocol Shortens “Door-to-Needle Time” and Improves Outcomes-Experience at a Tertiary Care Center in Qatar. J Stroke Cerebrovasc Dis. 2016 Aug; 25 (8): 2043-6.	RIHT: implementation of a protocol (“stroke code”, initiation of thrombolysis in the imaging room)
Huang Q, Song HQ, Ji XM, Cheng WY, Feng J, Wu J, Ma QF. Generalization of the Right Acute Stroke Prevention Strategies in Reducing in-Hospital Delays. PLoS One. 2016 May 6; 11 (5): e0154972.	RIHT: stroke protocol (pre-notification, simplification of image requests and analysis)
Puolakka T, Strbian D, Harve H, Kuisma M, Lindsberg PJ. Prehospital Phase of the Stroke Chain of Survival: A Prospective Observational Study. J Am Heart Assoc. 2016 May 2; 5 (5): e002808.	RPHT and RIHT: ambulance use and pre-hospital notification
Vidale S, Arnaboldi M, Bezzi G, Bono G, Grampa G, Guidotti M, Perrone P, Salmaggi A, Zarcone D, Zoli A, Agostoni E; Northern Lombardy Emergency Stroke Study Group. Reducing time delays in the management of ischemic stroke patients in Northern Italy. Int J Cardiol. 2016 Jul 15; 215: 431-4.	RPHT and RIHT: ambulance use and pre-hospital notification
Hsieh MJ, Tang SC, Chiang WC, Tsai LK, Jeng JS, Ma MH; Taipei EMS Stroke Collaborative Group. Effect of prehospital notification on acute stroke care: a multicenter study. Scand J Trauma Resusc Emerg Med. 2016 Apr 27; 24: 57.	RIHT: pre-hospital notification
Liang Z, Ren L, Wang T, Hu H, Li W, Wang Y, Liu D, Lie Y. Effective management of patients with acute ischemic stroke based on lean production on thrombolytic flow optimization. Australas Phys Eng Sci Med. 2016 Dec; 39 (4): 987-996.	RIHT: Implementation of a protocol (green pathway for stroke) and analysis/correction of the specific constraints of each “phase” from admission to the time of thrombolysis
Kim DH, Nah HW, Park HS, Choi JH, Kang MJ, Huh JT, Cha JK. Impact of Prehospital Intervention on Delay Time to Thrombolytic Therapy in a Stroke Center with a Systemized Stroke Code Program. J Stroke Cerebrovasc Dis. 2016 Jul; 25 (7): 1665-1670.	RPHT: ambulance use RIHT: direct communication from the pre-hospital to the stroke Via Verde
Advani R, Naess H, Kurz M. Mass Media Intervention in Western Norway Aimed at Improving Public Recognition of Stroke, Emergency Response, and Acute Treatment. J Stroke Cerebrovasc Dis. 2016 Jun; 25 (6): 1467-72.	RPHT: “mass media” campaigns. The duration of the effect is limited.
Dickson RL, Sumathipala D, Reeves J. Stop Stroke© Acute Care Coordination Medical Application: A Brief Report on Postimplementation Performance at a Primary Stroke Center. J Stroke Cerebrovasc Dis. 2016 May; 25 (5): 1275-1279.	RIHT: coordinated care protocol and smartphone application with stroke timer
Groot AE, van Schaik IN, Visser MC, Nederkoorn PJ, Limburg M, Aramideh M, de Beer F, Zwetsloot CP, Halkes P, de Kruijk J, Kruyt ND, van der Meulen W, Spaander F, van der Ree T, Kwa VI, Van den Berg-Vos RM, Roos YB, Coutinho JM. Association between i.v. thrombolysis volume and door-to-needle times in acute ischemic stroke. J Neurol. 2016 Apr; 263 (4): 807-13.	No differences depending on the volume of care provided by different hospitals
Moran JL, Nakagawa K, Asai SM, Koenig MA. 24/7 Neurocritical Care Nurse Practitioner Coverage Reduced Door-to-Needle Time in Stroke Patients Treated with Tissue Plasminogen Activator. J Stroke Cerebrovasc Dis. 2016 May; 25 (5): 1148-1152.	RIHT: Stroke patients supervised from admission by a neurocritical nurse
Rai AT, Smith MS, Boo S, Tarabishy AR, Hobbs GR, Carpenter JS. The ‘pit-crew’ model for improving door-to-needle times in endovascular stroke therapy: a Six-Sigma project. J Neurointerv Surg. 2016 May; 8 (5): 447-52.	RIHT (thrombectomy): protocol based on the concrete assignment of tasks to specific professionals and continuous monitoring
Choi PM, Desai JA, Kashyap D, Stephenson C, Kamal N, Vogt S, Bohm V, Suddes M, Bugbee E, Hill MD, Demchuk AM, Smith EE. Are All Stroke Patients Eligible for Fast Alteplase Treatment? An Analysis of Unavoidable Delays. Acad Emerg Med. 2016 Apr; 23 (4): 393-9.	IHTD: absence of pre-hospital notification; delay in registering the patient at the hospital, lack of equipment (imaging, monitoring)
Sadeghi-Hokmabadi E, Taheraghdam A, Hashemilar M, Rikhtegar R, Mehrvar K, Mehrara M, Mirnour R, Hassasi R, Aliyar H, Farzi M, Hasaneh Tamar S. Simple In-Hospital Interventions to Reduce Door-to-CT Time in Acute Stroke. Int J Vasc Med. 2016; 2016: 1656212.	RIHT: hospital pre-notification, prioritization of stroke patients for imaging
Kim DH, Bae HJ, Han MK, Kim BJ, Park SS, Park TH, Lee KB, Kang K, Park JM, Ko Y, Lee SJ, Choi JC, Kim JT, Cho KH, Hong KS, Cho YJ, Kim DE, Lee J, Lee J, Oh MS, Yu KH, Lee BC, Nah HW, Cha JK. Direct admission to stroke centers reduces treatment delay and improves clinical outcome after intravenous thrombolysis. J Clin Neurosci. 2016 May; 27: 74-9.	RPHT: direct admission to centers capable of intravenous thrombolysis
Sim J, Shin CN, An K, Todd M. Factors Associated With the Hospital Arrival Time in Patients With Ischemic Stroke in Korea. J Cardiovasc Nurs. 2016 Sep-Oct; 31 (5): E10-6.	RPHT: ambulance transport, emergency number contact
Madsen TE, Sucharew H, Katz B, Alwell KA, Moomaw CJ, Kissela BM, Flaherty ML, Woo D, Khatri P, Ferioli S, Mackey J, Martini S, De Los Rios La Rosa F, Kleindorfer D. Gender and Time to Arrival among Ischemic Stroke Patients in the Greater Cincinnati/Northern Kentucky Stroke Study. J Stroke Cerebrovasc Dis. 2016 Mar; 25 (3): 504-10.	RPHT: ambulance transport.
Itrat A, Taqui A, Cerejo R, Briggs F, Cho SM, Organek N, Reimer AP, Winners S, Rasmussen P, Hussain MS, Uchino K; Cleveland Pre-Hospital Acute Stroke Treatment Group. Telemedicine in Prehospital Stroke Evaluation and Thrombolysis: Taking Stroke Treatment to the Doorstep. JAMA Neurol. 2016 Feb; 73 (2): 162-8.	RPHT: ambulance with telemedicine and thrombolysis
Busby L, Owada K, Dhungana S, Zimmermann S, Coppola V, Ruban R, Horn C, Rochestie D, Khaldi A, Hormes JT, Gupta R. CODE FAST: a quality improvement initiative to reduce door-to-needle times. J Neurointerv Surg. 2016 Jul; 8 (7): 661-4.	RPHT: accelerated care protocol (pre-notification, direct referral to the imaging room, administration of thrombolysis in the imaging room)
Iglesias Mohedano AM, García Pastor A, García Arratibel A, Sobrino García P, Díaz Otero F, Romero Delgado F, Domínguez Rubio R, Muñoz González A, Vázquez Alen P, Fernández Bullido Y, Villanueva Osorio JA, Gil Núñez A. Factors associated with in-hospital delays in treating acute stroke with intravenous thrombolysis in a tertiary centre. Neurologia. 2016 Sep; 31 (7): 452-8.	IHTD: perform CT angiography, RIHT: activate stroke code:
Van Schaik SM, Scott S, de Lau LM, Van den Berg-Vos RM, Kruyt ND. Short Door-to-Needle Times in Acute Ischemic Stroke and Prospective Identification of Its Delaying Factors. Cerebrovasc Dis Extra. 2015 Jun 12; 5 (2): 75-83.	IHTD: absence of pre-notification; incorrect screening, imaging room unavailability, drug unavailability.
Sanossian N, Liebeskind DS, Eckstein M, Starkman S, Stratton S, Pratt FD, Koenig W, Hamilton S, Kim-Tenser M, Conwit R, Saver JL; FAST-MAG Investigators and Coordinators. Routing Ambulances to Designated Centers Increases Access to Stroke Center Care and Enrollment in Prehospital Research. Stroke. 2015 Oct; 46 (10): 2886-90.	RPHT: spatial reorganization of the pre-hospital emergency care network
Cerejo R, John S, Buletko AB, Taqui A, Itrat A, Organek N, Cho SM, Sheikhi L, Uchino K, Briggs F, Reimer AP, Winners S, Toth G, Rasmussen P, Hussain MS. A Mobile Stroke Treatment Unit for Field Triage of Patients for Intraarterial Revascularization Therapy. J Neuroimaging. 2015 Nov-Dec; 25 (6): 940-5.	RPHT and RIHT: imaging equipped ambulance, including for documentation of proximal occlusion
Chakraborty S, Ross J, Hogan MJ, Dowlatshahi D, Stotts G. Beating the clock: time delays to thrombolytic therapy with advanced imaging and impact of optimized workflow. J Stroke Cerebrovasc Dis. 2015 Jun; 24 (6): 1270-5.	RIHT (thrombectomy): total priority of the imaging room for the stroke patient, introduction of a protocol for the elaboration and quick readings of the images.
Kim A, Lee JS, Kim JE, Paek YM, Chung K, Park JH, Cho YJ, Hong KS. Trends in yield of a code stroke program for enhancing thrombolysis. J Clin Neurosci. 2015 Jan; 22 (1): 73-8.	RIHT: pre-notification, direct transfer from ambulance to imaging room, medicine bag
Wendt M, Ebinger M, Kunz A, Rozanski M, Waldschmidt C, Weber JE, Winter B, Koch PM, Freitag E, Reich J, Schremmer D, Audebert HJ; STEMO Consortium. Improved prehospital triage of patients with stroke in a specialized stroke ambulance: results of the pre-hospital acute neurological therapy and optimization of medical care in stroke study. Stroke. 2015 Mar; 46 (3): 740-5.	RPHT: “*mobile stroke unit*” (in urban contexts).
Wolters FJ, Paul NL, Li L, Rothwell PM; Oxford Vascular Study. Sustained impact of UK FAST-test public education on response to stroke: a population-based time-series study. Int J Stroke. 2015 Oct; 10 (7): 1108-14.	RTPH: mass education campaign
* Gray-marked cells correspond to studies that derive from interventions deliberately made to reduce pre- or in-hospital delay times, or to increase the number of patients benefiting from acute reperfusion therapies.	
